# KSHV oral shedding and plasma viremia result in significant changes in the extracellular tumorigenic miRNA expression profile in individuals infected with the malaria parasite

**DOI:** 10.1371/journal.pone.0192659

**Published:** 2018-02-09

**Authors:** Minako Ikoma, Soren Gantt, Corey Casper, Yuko Ogata, Qing Zhang, Ryan Basom, Michael R. Dyen, Timothy M. Rose, Serge Barcy

**Affiliations:** 1 Center for Global Infectious Disease Research, Seattle Children’s Research Institute, Seattle, Washington, United States of America; 2 Department of Pediatrics, University of British Columbia, BC Children’s Hospital, Vancouver, British Columbia, Canada; 3 Infectious Disease Research Institute, Seattle, Washington, United States of America; 4 Departments of Medicine and Global Health, University of Washington, Seattle, Washington, United States of America; 5 Proteomics, Fred Hutchinson Cancer Research Center, Seattle, Washington, United States of America; 6 Genomics, Fred Hutchinson Cancer Research Center, Seattle, Washington, United States of America; 7 Department of Pediatrics, University of Washington, Seattle, Washington, United States of America; Institut de Pharmacologie Moleculaire et Cellulaire, FRANCE

## Abstract

Kaposi's sarcoma herpesvirus (KSHV) is the etiological agent of Kaposi’s sarcoma (KS). Both KSHV and HIV infections are endemic in Uganda, where KS is among the most common cancers in HIV-infected individuals. Recent studies examined the use of small RNAs as biomarkers of disease, including microRNAs (miRNAs), with viral and tumor-derived miRNAs being detected in exosomes from individuals with KSHV-associated malignancies. In the current study, the host and viral extracellular mature miRNA expression profiles were analyzed in blood of KS-negative individuals in Uganda, comparing those with or without KSHV detectable from the oropharynx. We observed increased levels of cellular oncogenic miRNAs and decreased levels of tumor-suppressor miRNAs in plasma of infected individuals exhibiting oral KSHV shedding. These changes in host oncomiRs were exacerbated in people co-infected with HIV, and partially reversed after 2 years of anti-retroviral therapy. We also detected KSHV miRNAs in plasma of KSHV infected individuals and determined that their expression levels correlated with KSHV plasma viremia. Deep sequencing revealed an expected profile of small cellular RNAs in plasma, with miRNAs constituting the major RNA biotype. In contrast, the composition of small RNAs in exosomes was highly atypical with high levels of YRNA and low levels of miRNAs. Mass spectrometry analysis of the exosomes revealed eleven different peptides derived from the malaria parasite, *Plasmodium falciparum*, and small RNA sequencing confirmed widespread plasmodium co-infections in the Ugandan cohorts. Proteome analysis indicated an exosomal protein profile consistent with erythrocyte and keratinocyte origins for the plasma exosomes. A strong correlation was observed between the abundance of Plasmodium proteins and cellular markers of malaria. As *Plasmodium falciparum* is an endemic pathogen in Uganda, our study shows that co-infection with other pathogens, such as KSHV, can severely impact the small RNA repertoire, complicating the use of exosome miRNAs as biomarkers of disease.

## Introduction

Kaposi's sarcoma herpesvirus (KSHV), also known as human herpesvirus-8 (HHV-8), is the etiological agent of Kaposi’s sarcoma (KS) and other AIDS-associated malignancies, including primary effusion lymphoma (PEL) and subtypes of multicentric Castleman disease (MCD) [1, 2]. In Uganda, where both KSHV and HIV infections are endemic, the incidence of KS is high, making it among the most common forms of cancer among HIV-infected persons [3, 4].

Diagnosis of KSHV infection is challenging, particularly in individuals without clinical signs of KSHV-associated diseases. Typically, exposure to a herpesvirus infection is associated with a persistent virus-specific antibody response. However, serological tests for KSHV infection are hampered by poor sensitivity and sero-reversion [5]. In KSHV-infected individuals, the humoral immune response to infection can decline to the extent that the individual could appear seronegative, even though carrying a latent infection. Therefore, many studies utilize the detection of KSHV in biologic specimens (most commonly plasma, peripheral blood mononuclear cells, or saliva) by qPCR to examine explore correlations between viral genome replication and the clinical or biologic correlates of disease. Several studies have shown that the detection rate for KSHV DNA is highest in the oropharynx [6–8]. Since KSHV is intermittently detected in the oral epithelium, and the sensitivity of antibody assays for diagnosis of HHV-8 is limited, detection of KSHV by qPCR from consecutive oral swabs may be the most reliable method to diagnose KSHV infection in asymptomatic individuals [9, 10]. Recently, it was suggested that the molecular content of exosomes in blood could be used as a diagnostic biomarker for KSHV-associated malignancies, but the relevance for diagnosis of infection in KS-negative individuals was not assessed [11]. Another study suggested that detection of KSHV-encoded small RNAs by RT-qPCR could be used to improve KSHV diagnosis [12].

The cellular and viral mechanisms by which KSHV infection progresses to malignancy in some individuals remain unclear. The KSHV genome encodes several oncogenic proteins with the potential to induce a malignant phenotype in infected cells [13, 14]. Although some of these genes are expressed during viral lytic replication, their messenger RNA (mRNA) transcripts are also detected during latency in certain cell types [15, 16]. Experimental observations support a model for KSHV oncogenesis in which infected cells expressing oncogenic viral proteins promote angiogenesis and cell proliferation in autocrine and paracrine fashions [17, 18]. KSHV carcinogenesis is also linked to the activity of small RNA transcripts encoding microRNAs (miRNA) [19, 20]. MiRNAs are short non-coding RNAs that have modulatory effects on different signaling pathways through repression of translation initiation of specific messenger RNAs (mRNAs)[21]. Those miRNAs targeting host genes associated with carcinogenesis, including oncogenes and tumor suppressors, are called oncomiRs [22, 23]. Several thousand cellular miRNAs have been identified in the human genome [24] and viral miRNAs have been identified in KSHV and other members of the herpesvirus family infecting humans, including Epstein-Barr virus (EBV) [25]. The KSHV genome encodes more than 20 different viral miRNAs, including some known to have oncomiR activity [26–28]. The regulatory activity of miRNAs is not only autocrine in the expressing cell but can be paracrine as miRNAs are released from cells and can persist in stable forms circulating in bodily fluids including plasma. To prevent rapid degradation, miRNAs are either packaged in microvesicles called exosomes or loaded into lipoproteins, including high and low density lipoproteins (HDL, LDL). Exosomes and lipoproteins transport and deliver miRNAs to distant cells where the miRNAs can function to regulate gene expression [29, 30]. This suggests that extracellular miRNAs could promote KSHV carcinogenesis in a paracrine manner [31].

The molecular composition of exosomes reflects the cell type of origin and has shown promising potential as a biomarker in disease diagnostic. The exosomal protein and miRNAs composition differs between exosomes released by healthy and pathogen-infected cells [32]. Following viral or parasitic infection, cells release extracellular vesicles with distinct molecular repertoires in easily accessible bodily fluids such as plasma [33]. A recent study confirmed the existence of a specific exosomal miRNA expression signature composed of human and KSHV encoded oncogenic miRNAs, consistently expressed in plasma exosomes from patients presenting with KS or PEL malignancies [11]. Other in vitro studies revealed distinct miRNA expression repertoires associated with KS tumors, KSHV infected cells and progressive stages of endothelial cell transformation, suggesting a biological relevance for different miRNA expression profiles in KSHV tumorigenesis [34]. These previous studies had in common the characterization of cellular and/or viral miRNAs in either tumor microenvironments or in patients with a pre-existing malignancy.

*Plasmodium falciparum*, the parasite responsible for malaria, is also endemic in Uganda. The incidence of KS is the highest in sub-Saharan African countries where malaria is endemic (Semeere, Busakhala et al. 2012). Recent studies revealed that changes to the host miRNA signatures in human patients with malaria were associated with disease severity. The extent to which *Plasmodium falciparum* infection can change the small RNA repertoire in plasma exosomes isolated from individuals with either uncomplicated or asymptomatic malaria infection is still unknown. Although malaria has been proposed as a risk factor for KSHV infection, no evidence of molecular interactions between the KSHV and Plasmodium pathogens has been reported (Thakker and Verma 2016). Furthermore, it is unknown how the small RNA repertoire is altered during co-infections of these pathogens.

In the current study, we examined the cellular and viral miRNA expression profile in KSHV-infected people without clinical signs of KSHV-associated disease. We observed specific miRNA profiles in plasma of individuals with oral shedding of KSHV DNA. We detected different cellular miRNAs circulating in plasma of KSHV-infected individuals with or without HIV co-infection and identified changes in miRNA expression after anti-retroviral therapy. In addition, we characterized the viral miRNAs encoded by KSHV and EBV during natural infections in these individuals. Our data suggest that miRNAs function as mediators in a paracrine signaling mechanism promoting KSHV malignancies. Finally, we show that co-infection with the malaria parasite is associated with major alterations in the content of plasma exosomes limiting the use of exosomal miRNAs as biomarkers for KSHV infection and disease in individuals with polymicrobial infections.

## Results

### Cohort participants

We collected blood samples and oral swabs from three different cohorts of adults in Uganda. Importantly, none of the individuals enrolled were diagnosed with Kaposi’s sarcoma (KS) at the time of collection. Oral swabs were collected daily for a shedding period of 15 consecutive days and plasma was isolated from blood draws collected weekly during the same study period. The first cohort (KSHV+/HIV-) consisted of 8 individuals that tested PCR positive for KSHV oral shedding, but were serologically negative for HIV. The second cohort (KSHV+/HIV+) consisted of 28 individuals that tested positive for both KSHV and HIV. These individuals were part of a study testing the effects of antiretroviral therapy (ART) on KSHV replication and disease progression. In addition to testing positive for KSHV and HIV, these study participants were ART-naïve with a minimum absolute CD4+ T count of 250 cells. Oral swabs and plasma samples were collected prior to the start of antiretroviral therapy treatment and after two years of treatment. The absolute CD4+ T cell count and HIV viral loads were determined at both time points. The third cohort (KSHV-/HIV-) consisted of 19 individuals tested HIV-seronegative and consistently PCR negative for KSHV in oral swabs collected during the study period. We considered this cohort as reference in our analysis. Although not actively shedding during the study period, individuals in this cohort could still be infected since KSHV is endemic in Uganda. Because KSHV infection is characterized by episodes of sporadic viral DNA detection in oral swab and plasma samples, the significance of a viral load measured at a single time point is limited. Thus, for each individual we also calculated a rate of detection. The rate of detection in oral swabs is the percentage of samples in which KSHV is detected by qPCR in oral swabs collected over the 15 consecutive day period. Similarly, the rate of KSHV detection in plasma is the percentage of samples in which KSHV is detected by qPCR in plasma collected weekly during the same screening period (day 0, day 7, and day 15).

### Increased levels of oncogenic miRNAs and decreased levels of tumor suppressor miRNAs are observed in plasma from individuals with detectable KSHV oral shedding

Initially, we characterized the miRNA expression profiles in plasma from groups of three individuals randomly selected from each cohort (Table 1). Mature miRNAs were detected using the nCounter miRNA expression assay based on the NanoString screening platform (S1 Table). The average age was 38 (median 38, range: 34–44) in the KSHV-infected group (KSHV+/HIV-) and 31 (median 27, range: 27–41) in the KSHV+/HIV+ co-infected group. In the KSHV-/HIV- group, the average age was 35 (median 37, range 28–41). Before ART therapy, the average CD4+ T cell count for the KSHV+/HIV+ co-infected group was 273 (median 268, range 267–285) and the average HIV viral load was 5.37 log_10_ copies/ml (median = 5.12, range: 4.89–5.7). As seen in Table 1, the rate of KSHV detection in oral swabs was higher in the KSHV+/HIV+ group compared to KSHV+/HIV- group (average: 97% vs 17%) in agreement with previous observations. Following two years of ART therapy, all three HIV-infected people showed a significant increase in their blood CD4+ T cell count and a substantial decrease in their HIV viral load, indicating a positive response to the ART regimen.

**Table 1:** Patient demographics and viral status.

Viral screening	KSHV-^a^/HIV-			KSHV+/HIV-			KSHV+/HIV+					
Patient ID	**338–0**	**358–9**	**364–9**	**144–1**	**146–6**	**169–3**	**25–1**		**27–5**		**131–6**	
Age (year)	37	28	41	34	38	44	27		41		27	
Gender	F	F	F	M	M	M	F		M		M	
ART status	N.A.^f^	N.A.	N.A.	N.A.	N.A.	N.A.	Pre	Post^**b**^	Pre	Post	Pre	Post
CD4+ T cell count	N.A.	N.A.	N.A.	N.A.	N.A.	N.A.	267	487	268	558	285	858
HIV viral load	N.A.	N.A.	N.A.	N.A.	N.A.	N.A.	5*10^5^	6.5*10^3^	7,9*10^4^	<20	1,3*10^5^	<20
KSHV in oral swab	N.D.^g^	N.D.	N.D.	2,5*10^5^	1,5*10^6^	N.D.	1,4*10^3^	2,6*10^5^	2,9*10^4^	N.D.	1,6*10^5^	2,7*10^3^
Rate of shedding (%) ^**c**^	0%	0%	0%	20%	20%	13%	93%	100%	100%	6%	100%	13%
KSHV plasma viral load ^e^	N.D.	N.D.	N.D.	N.D.	N.D.	N.D.	N.D.	N.D.	N.D.	N.D.	N.D.	54
Rate of plasma viremia^**d**^	0%	0%	0%	0%	0%	0%	60%	0%	0%	0%	0%	66%

^a^ KSHV- = consistently tested negative by specific qPCR on oral swab and plasma samples during the screening period.

^b^Post Art time point is at the end of 2 years of anti-retroviral treatment.

^**c**^Rate of oral shedding or rate of plasma viremia were calculated as the percentage of qPCR positive days during the screening session.

^d^Plasma samples were collected weekly (day0, day7, day15) during the screening session.

^e^ viral load (viral genome copies/ml) measured for samples collected on day o during the screening session.

^f^NA = not available.

^g^N.D. = below detection level.

**Fig 1:**
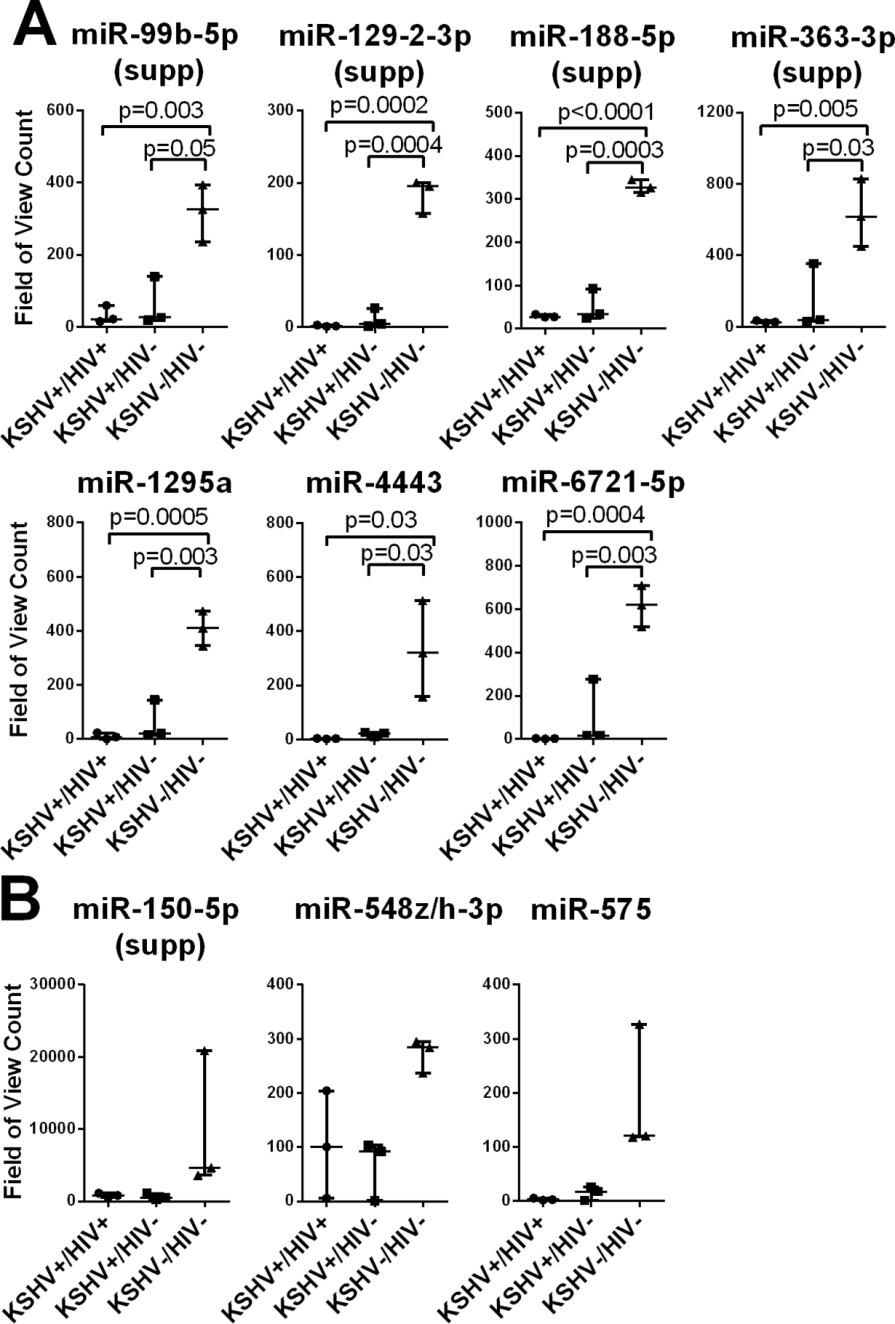
Plasma levels of tumor suppressor miRNAs are decreased in individuals with detectable KSHV oral shedding. The expression levels of cellular miRNAs in total RNA isolated from plasma were determined using the NanoString microRNA platform. MiRNAs with significant changes in expression among the KSHV-/HIV-, KSHV+/HIV-, and KSHV+/HIV+ groups were identified using the Kruskal-Wallis statistical test. MiRNAs with known tumor suppressor (supp) activity are indicated. MiRNAs with (A) or without (B) a significant changes in level of expression when comparing the groups of individuals pairwise are shown. P values indicate were calculated using a t-test.

Accumulating evidence suggests that a single miRNA can regulate the expression of various cellular proteins, some of which may have opposing oncogenic or tumor-suppressive functions. The effect of miRNA expression is likely the result of a complex network of interactions dictated by a specific environment. As such, miRNA expression can be associated with contrasting effects depending on the context [35]. Following data analysis, 26 miRNAs showed significant differential expression within the three groups. The majority of these miRNAs had either tumor suppressive (8/26, supp) or oncogenic (13/26, onco) activities. Seven of the 26 miRNAs, miR-99b-5p, miR-129-3p, miR-188-5p, miR-363-3p, miR-1295a, miR-4443 and miR-6721-5p, showed significantly decreased expression in individuals with detectable oral shedding (KSHV+/HIV- and KSHV+/HIV+ groups) compared to individuals without detectable oral shedding (KSHV-/HIV- group), regardless of HIV status (Fig 1A). Four of these, miR-99b-5p, miR-129-3p, miR-188-5p, and miR-363-3p, have known tumor suppressor activities. Three other miRNAs, miR-150-3p, miR-548z/h-5p, and miR-575, also showed decreased expression in both groups with detectable oral shedding (KSHV+/HIV-, KSHV+/HIV+)(Fig 1B), but the differences were not statistically significant due to greater variability in their expression levels. One of these miRNAs, miR-150-5p, has known tumor suppressor activity [36, 37].

**Fig 2:**
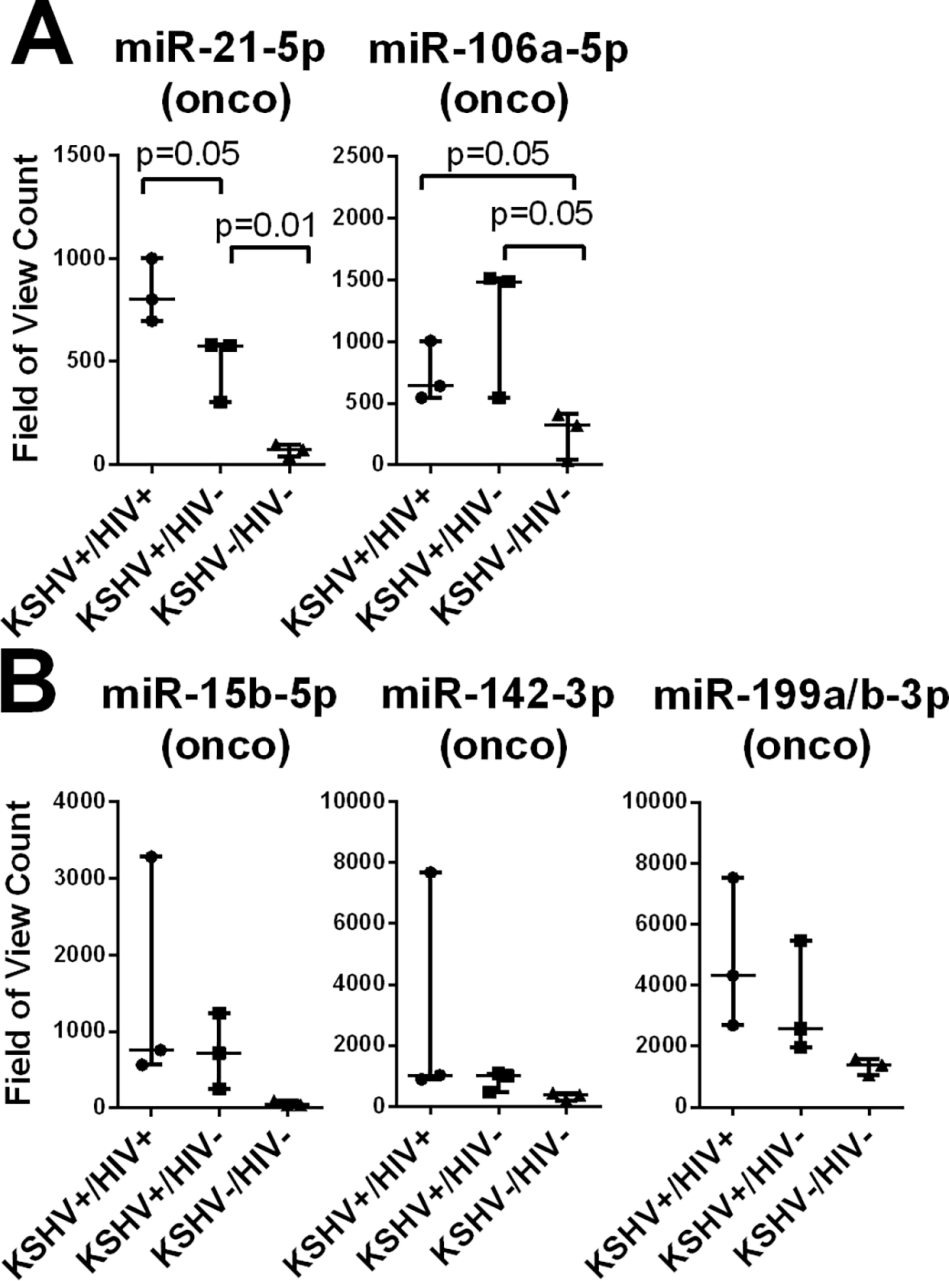
Plasma levels of oncogenic miRNAs are increased in individuals with detectable KSHV oral shedding. The expression levels of cellular miRNAs in total RNA isolated from plasma were determined using the NanoString microRNA platform. MiRNAs with significant changes in expression among the KSHV-/HIV-, KSHV+/HIV-, and KSHV+/HIV+ subgroups were identified using the Kruskal-Wallis statistical test. MiRNAs with known oncogenic (onco) activity are indicated. MiRNAs with (A) or without (B) a significant changes in level of expression when comparing the groups of individuals pairwise are shown. P values indicate were calculated using a t-test.

In contrast, two of the 26 miRNAs, miR-21-5p and miR-106a-5p, showed significantly increased expression in both groups with detectable oral shedding (KSHV+/HIV- and KSHV+/HIV+) compared to group without (KSHV-/HIV-) regardless of HIV status (Fig 2A). Both of these miRNAs have known oncogenic activities [38, 39]. In addition, the expression level of the oncogenic miR-21-5p was significantly higher in the KSHV+/HIV+ group compared to the KSHV+/HIV- group. Three additional miRNAs, including miR-15b-5p, miR-142-3p and miR-199a/b-3p showed increased expression in individuals with detectable oral shedding of KSHV (KSHV+/HIV- and KSHV+/HIV+) compared to the group without detectable oral shedding (KSHV-/HIV-), regardless of HIV status (Fig 2B), but the differences were not statistically significant due to a greater variability in their expression levels.

Thus, in KSHV-infected individuals without KS, the presence of detectable levels of KSHV DNA in the oral epithelium is associated with an upregulated expression of oncogenic miRNAs and downregulated expression of tumor suppressor miRNAs in plasma, suggesting that oral shedding in a KSHV-infected individual could be associated with a higher risk of developing a KSHV-associated malignancy. In addition, this pattern of pro-tumorigenic miRNA expression precedes one or more KSHV oral shedding episodes during the subsequent two week period and is absent from the plasma of individuals with no evidence of KSHV oral shedding.

### The pro-tumorigenic pattern of cellular miRNAs expression observed in KSHV-infected individuals is exacerbated in study participants co-infected with HIV

**Fig 3:**
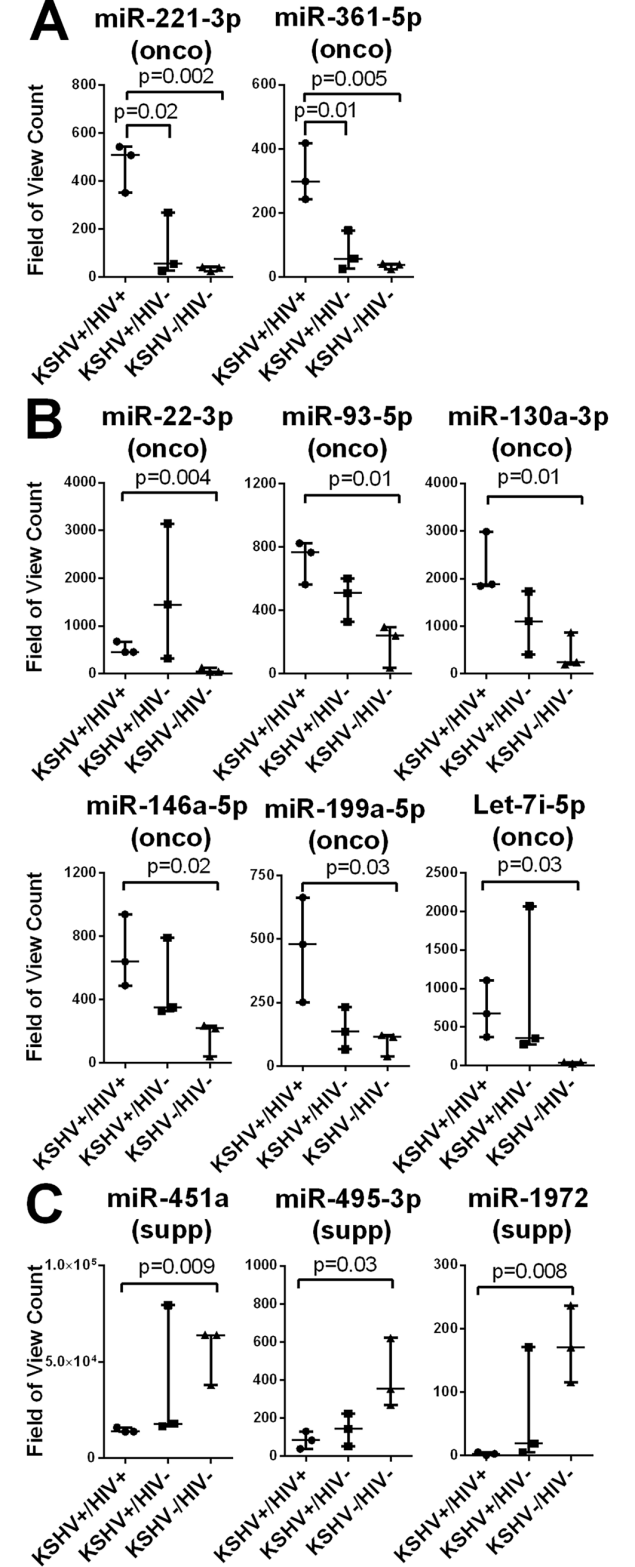
Changes in tumorigenic miRNAs level of expression in plasma are exacerbated in individuals dually-infected with KSHV and HIV. The expression levels of cellular miRNAs in total RNA isolated from plasma were determined using the NanoString microRNA platform. MiRNAs with significant changes in expression among the KSHV-/HIV-, KSHV+/HIV-, and KSHV+/HIV+ subgroups were identified using the Kruskal-Wallis statistical test. MiRNAs with known oncogenic (onco) or tumor suppressor (sup) activities are indicated. Oncogenic miRNA with significant differential expression between the KSHV+/HIV-, and KSHV+/HIV+ groups (A), or the KSHV-/HIV-, KSHV+/HIV+ groups (B) are shown. Tumor suppressor miRNAs with significant differential expression between the KSHV-/HIV-, KSHV+/HIV+ groups are shown separately. P values indicate were calculated using a t-test.

The remaining 11 of 26 differently expressed cellular miRNAs showed significant differences in the HIV co-infected group compared to the HIV-negative groups. The levels of two miRNAs, miR-221-3p, and miR-361-5p, were significantly increased in the KSHV+/HIV+ co-infected group compared to both of the HIV-negative groups (KSHV+/HIV- and KSHV-/HIV-) (Fig 3A). Both of these upregulated miRNAs have known oncogenic activity. Six miRNAs, miR22-3p, miR-93-5p, miR-130a-3p, miR-146a-5p, miR-199a-5p and Let-7i-5p, were significantly increased in the KSHV+/HIV+ co-infected group compared to KSHV-/HIV- group (Fig 3B). All six of these miRNAs have known oncogenic activity. The median level of all of these miRNAs, except miR-22-3p, was higher in KSHV+/HIV+ co-infected group than in the KSHV+/HIV- group, although this did not reach a level of significance due to variability between individuals. The levels of three miRNAs, miR-451a, miR-495-3p and miR-1972, were significantly decreased in the KSHV+/HIV+ group compared to the KSHV-/HIV- group (Fig 3C). All three of these miRNAs have known tumor suppressor activity. The median level of all of these miRNAs was lower in the KSHV+/HIV+ co-infected group compare to the KSHV+/HIV- group, although this did not reach significance.

Thus, our results suggest that the pro-tumorigenic pattern of cellular miRNAs expression observed in KSHV-infected individuals without KS is exacerbated in study participants co-infected with HIV. This observation supports previous studies which reported enhanced progression and development of KSHV-associated tumors in HIV co-infected individuals.

### ART therapy reverses the effects of HIV co-infection on cellular oncomiR expression

Our results indicated that alterations of the cellular miRNA repertoire in plasma of KSHV-infected individuals were often amplified by HIV co-infection. Therefore, we investigated whether or not suppression of HIV viral loads and rebound in CD4+ T cell count following ART therapy impacted the cellular miRNA profile in plasma. The nCounter miRNA expression multiplexed assay was used to profile the miRNA expression pattern in the three KSHV+/HIV+ co-infected individuals before and 2 years after initiation of ART. The drug regimen consisted of a combination of nucleotide, nucleoside, and non-nucleoside reverse transcriptase inhibitors (lamivudine, tenofovir and efavirenz). All three individuals responded to the treatment as attested by a rebound in their absolute CD4+ T cell number and a strong reduction in their HIV viral loads in plasma (Table 1).

**Fig 4:**
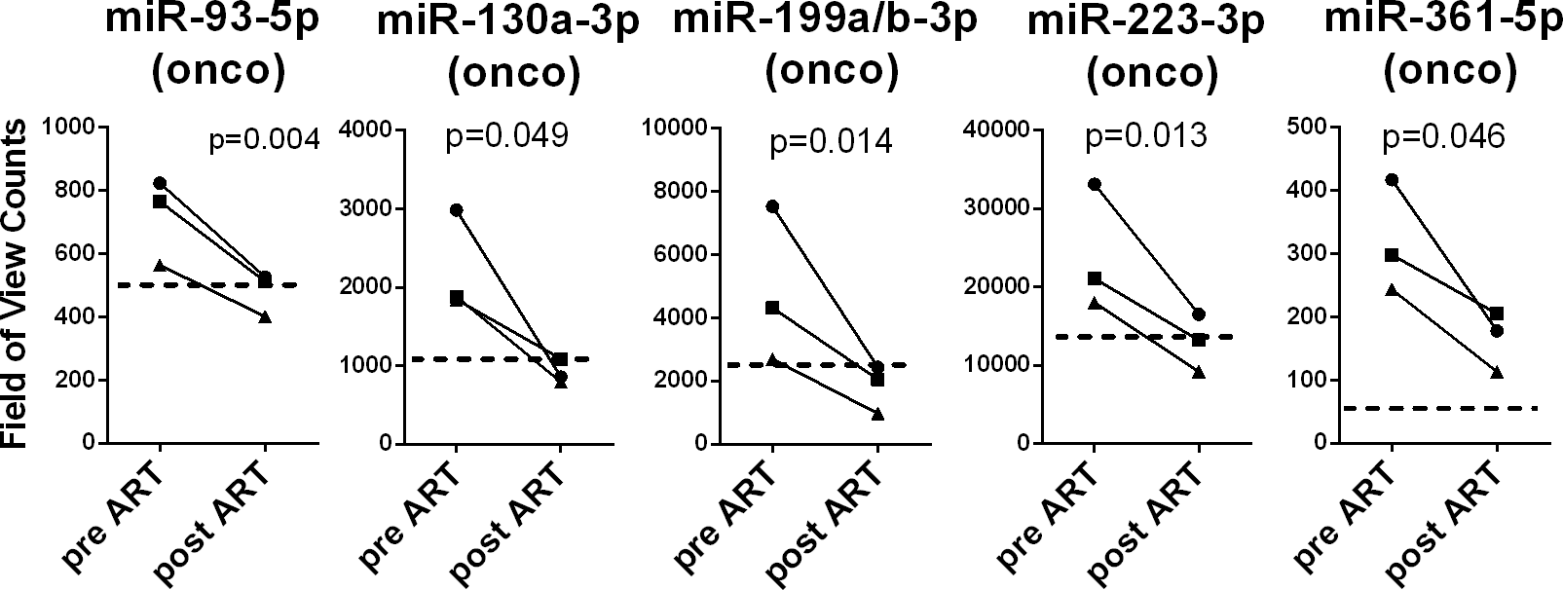
MiRNA levels of expression in HIV infected patients before and after ART treatment. MicroRNAs levels of expression were determined using the nanoString microRNA platform on total RNA isolated from plasma samples collected before and after 2 years of ART therapy. P values indicated were calculated using a ratio paired t-test. The respective level of expression measured in KSHV+/HIV- individuals (median values) is shown in dashed line.

For each KSHV +/HIV+ co-infected individual, we compared the miRNA plasma levels before and after ART. As shown in Fig 4, five oncogenic miRNAs, including miR-93-5p, miR-130a-3p, miR-199a/b-3p, miR-223-3p, and miR-361-5p, showed a significant decrease in plasma levels in each patient following ART, down to levels seen in KSHV+/HIV- individuals. Four of these 5 miRNAs (miR-93-3p, miR-130a-3p, miR-199a/b-3p, and miR-361-5p) were in the group of miRNAs initially identified as differentially expressed in the three original cohorts. These miRNAs showed significantly higher plasma levels in the KSHV+/HIV+ co-infected group compared to both group without HIV (KSHV+/HIV- and KSHV-/HIV-) (Figs 1 and 3). The median levels of the fifth miRNA, miR-223-3p was higher in the co-infected group compared to the HIV negative groups, but this increase was not statistically significant due to a greater variability between samples (median:13998 for the KSHV+/HIV- group vs 21073 for the KSHV+/HIV+ group). Expression levels for the remaining miRNAs identified above as differently expressed in KSHV+/HIV+ co-infected individuals were not affected by ART.

Thus, our results show that ART therapy could decrease the levels of specific oncogenic miRNAs that are upregulated in individuals co-infected with KSHV and HIV. Since these miRNAs are associated with oncogenic activity, decreased expression following ART could correlate with a decreased risk of KSHV-associated AIDS-related malignancies.

### RNAseq analysis reveals similar distributions of small cellular RNA species in plasma of KSHV-infected individuals with or without HIV co-infection

MiRNAs are only one of several known small RNA biotypes present in plasma. To comprehensively examine the distribution pattern of small RNA biotypes, we performed high throughput RNA sequencing (RNAseq) of total small RNAs purified from plasma.

Plasma samples from five individuals were randomly selected from each of the KSHV+/HIV+ and KSHV+/HIV- cohorts were identified for RNAseq analysis (Table 2).

**Table 2:** Patient demographics and viral status.

	KSHV+/HIV-					KSHV+/HIV+^a^				
Patient’s ID	**138–9**	**146–6**	**169–3**	**361–0**	**365–1**	**033–1**	**091–1**	**131–6**	**174–5**	**181–8**
Age (year)	57	38	44	42	37	26	40	27	35	26
Gender	M	M	M	M	F	M	M	M	M	F
CD4+ count	N.A.^e^	N.A.	N.A.	N.A.	N.A.	355	303	285	348	331
HIV viral loads	N.A.	N.A.	N.A.	N.A.	N.A.	2,5*10^5^	1,6*10^5^	1,3*10^5^	1,7*10^5^	1*10^4^
KSHV in oral swab^**d**^	9,6*10^4^	1,5*10^5^	N.D.^f^	N.D.	3,9*10^5^	N.D.	2,5*10^5^	1,6*10^5^	4,4*10^5^	1,2*10^3^
Rate of oral shedding^b^	100%	13%	7%	0%	30%	13%	100%	100%	93%	33%
KSHV plasma viremia^**d**^	885	N.D.	N.D.	N.D.	N.D.	620	164	N.D.	2,3*10^5^	1*10^3^
Rate of plasma viremia^c^	66%	33%	0%	33%	0%	100%	100%	0%	66%	33%

^a^Blood samples from HIV patients were collected prior to start ART treatment.

^b^Rate of oral shedding or rate of plasma viremia calculated as the percentage of qPCR positive days over the screening session.

^c^Plasma samples were collected weekly (day0, day7, day15) during the screening session.

^d^ viral load (viral genome copies/ml) measured for samples collected on day 0 during the screening session

^e^NA = not available,

^f^N.D. = below detection level

The number of individuals per group was chosen to account for inherent variability associated with human samples when using high throughput sequencing. Since large volumes of plasma were needed to isolated sufficient quantities of small RNAs for high throughput sequencing, only plasma samples with sufficient sample size were chosen for analysis. Participants included both men (80%) and women (20%). The mean age was 43 (median 42, range: 37–57) among the KSHV-infected group and 30 (median: 27, range: 26–40) among the KSHV/HIV co-infected group. The mean CD4+ T cell count among the HIV infected individuals was 324 cells /uL (median: 331, range: 365–385) and the HIV viral load mean was 5.16 log_10_ copies /ml (median: 5.2, range: 4.02–5.4). All samples from HIV infected individuals were collected before initiating ART therapy, on the first day of the screening period. As seen for the groups of individuals in the previous analysis, the average KSHV detection rate in oral swabs across the 15 day screening period was higher in the KSHV+/HIV+ group than in the KSHV+/HIV- group (average: 68% vs 30%). Similarly, the average KSHV detection rate in plasma was 60% in the KSHV+/HIV+ group compared to 26% in KSHV+/HIV- group.

**Fig 5:**
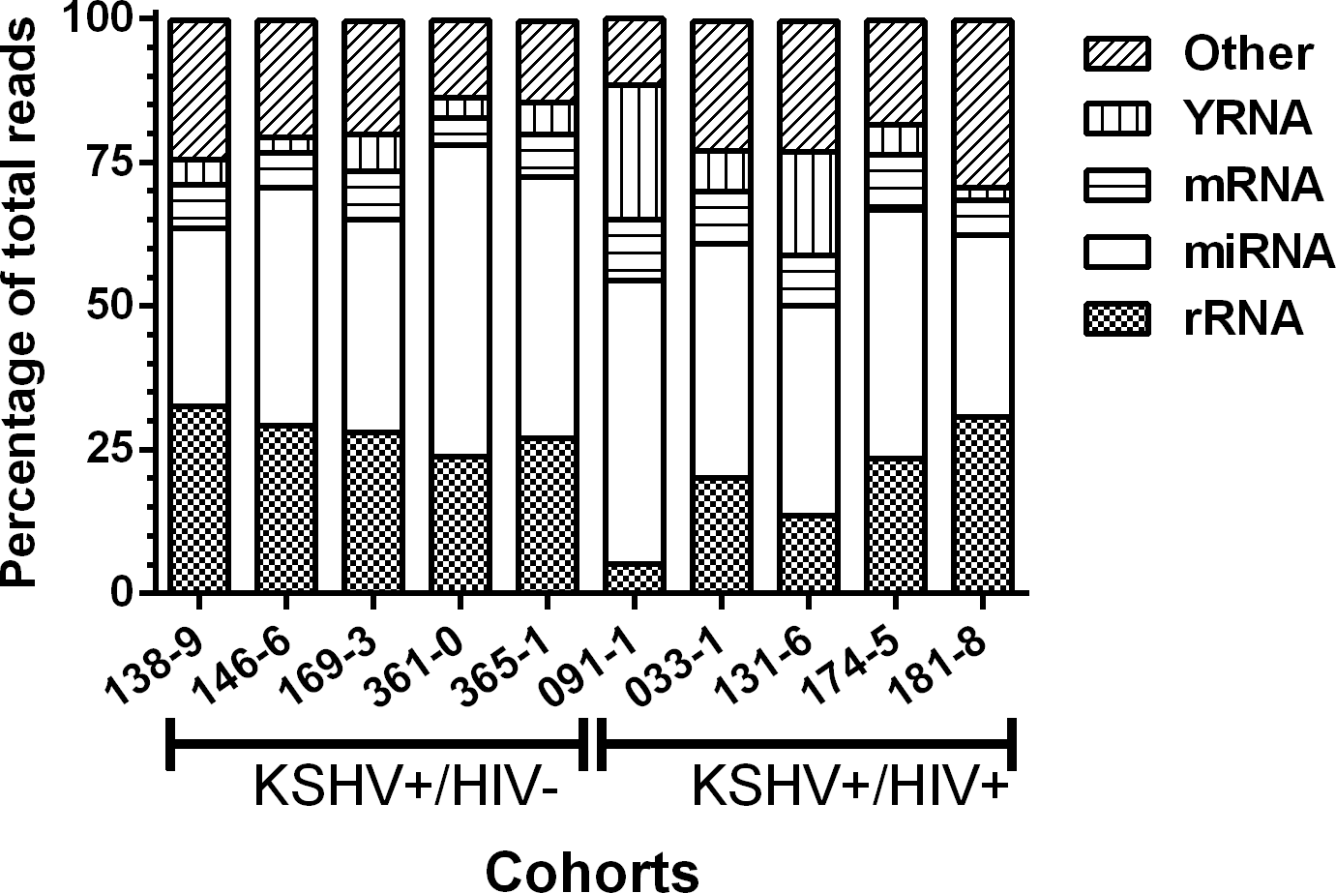
Small RNA biotypes distribution in plasma. Segments of the bar indicate the percent of reads attributed to each RNA type among all the small RNA reads that mapped to the human reference genome (hg19).

For each plasma sample, a library of cDNA copies of small RNAs with lengths ranging from 11–29 nucleotides (nt) were deep sequenced with more than 14 million quality filtered reads per library. The percentage of reads mapping specifically to ribosomal RNA (rRNA), transfer RNA (tRNA), miRNA (microRNA), piwi-RNA (piRNA), snoRNA, YRNA, and protein-coding mRNAs were determined (Fig 5). The distribution of reads for each RNA biotype was very consistent among samples from the KSHV+/HIV- group with correlation coefficients ranging from 0.9 to 1.0; (p = 0.08–0.01) More variability was observed in the KSHV+/HIV+ group with correlation coefficient ranging from 0.1 to 0.9 (p = 0.95–0.01).

In agreement with previously published data, the most abundant type of small RNAs in plasma was the miRNA, representing on average 41% of the total mapped reads (median = 41, range 31–54). Ribosomal RNA was also abundantly detected, accounting for an average of 23% of the mapped reads (median = 25, range 5–32). As expected from previous reports, reads mapped to YRNA sequences accounted for an average of 7% of the total mapped reads (median = 57, range 2–23). Finally, reads mapped to protein-coding mRNAs constituted an average of 7% of the total number of mapped reads (median = 7, range 4–10). Three remaining RNA biotypes, including piwiRNA, snoRNA, and tRNA represented only minor populations of RNAs in plasma with less than 1% of the total reads mapping to each of these biotype. Our analysis showed that the relative representation of extracellular small RNA biotypes in plasma was similar between the KSHV+/HIV- and KSHV+/HIV+ groups although the KSHV+/HIV+ group showed grater variability.

### RNAseq analysis identified differently expressed oncomiRs in plasma from KSHV+/HIV+ co-infected individuals

To extend and validate our initial Nanostring analysis of the plasma miRNAs, the miRNA reads from the high throughput sequencing data sets were aligned to reference sequences available from a database of cellular miRNAs (miRBase21). The abundance of reads in our datasets allowed us to identify up to 385 different mature miRNAs in the plasma samples using a filtered minimum read count of ten. The DESeq2 software was used to normalize the read counts associated with each mature miRNA and perform a differential expression analysis between the KSHV-infected group and the KSHV+/HIV+ co-infected group. Using the DESeq 2 software we identified 22 miRNAs predicted to be differentially expressed between KSHV-infected individuals with and without HIV co-infection.

**Fig 6:**
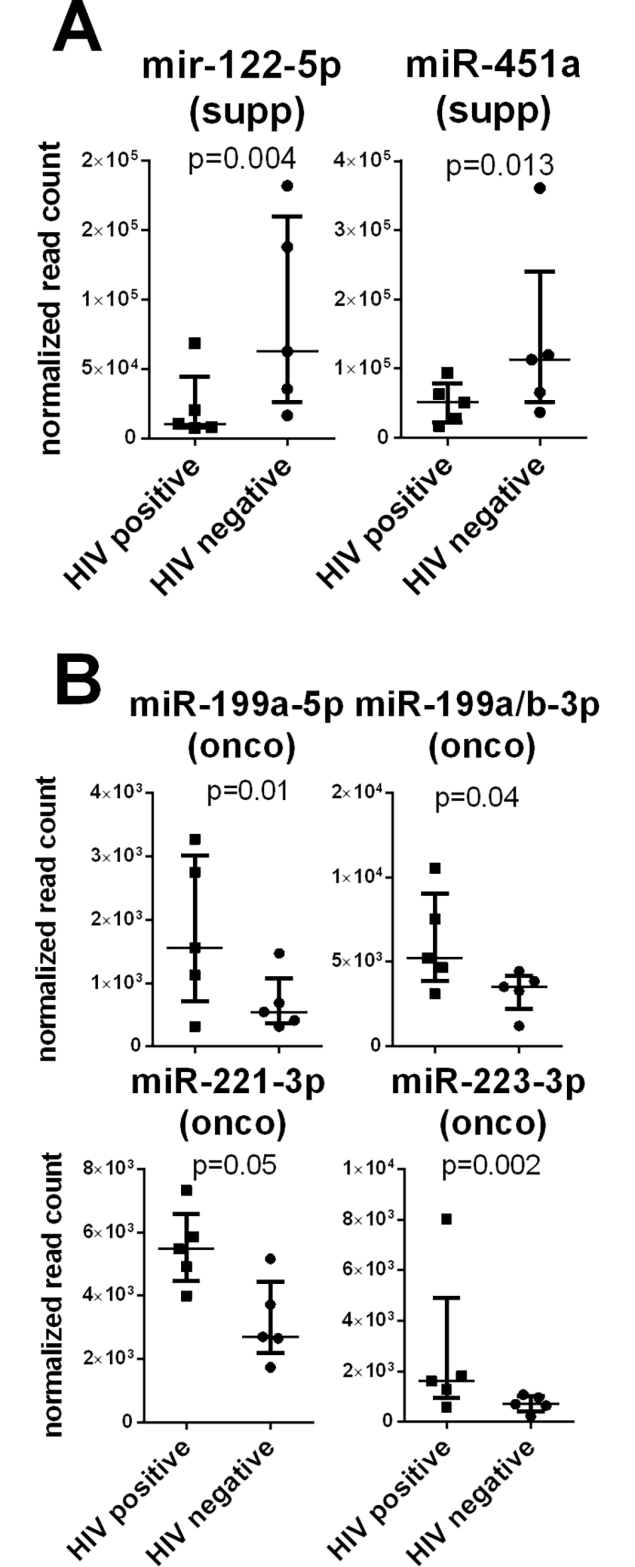
MiRNAs differently expressed between KSHV+/HIV- and KSHV+/HIV+ groups of people. Sequencing reads from libraries of purified small RNAs were mapped against a database of human miRNA sequences (miRBase 21). Data were normalized and analyzed for differential expression using the DESEq2 software. MiRNAs with a significant decrease (A) or increase (B) in plasma level between KSHV-infected individuals with (HIV+) or without HIV co-infection (HIV-) are shown.

The differential expression for one of them, miR-122-5p, was statistically significant after controlling for false discovery rate (FDR) (p = 0.004, adjusted p = 0.0852) (Fig 6A) indicating that the HIV status had a limited effect on the plasma miRNA repertoire expression. Four miRNAs, including miR-451a, miR-221-3p, miR-199a-5p, and miR-199a/b-3p, had been previously identified as differentially expressed in a different group of individuals from the same infection cohort using the Nanostring platform. Of these, miR-451, a known tumor suppressor, was decreased in the HIV-infected group (Fig 6A), while miR-221-3p, miR-199a-5p and miR-199a/b-3p, all known oncogenes, were increased in the HIV-infected group (Fig 6B). Another miRNA initially identified by the DESeq2 software was the oncogenic miR-223-3p (Fig 6B), which also showed previously a significantly decrease expression level after ART treatment.

While the RNAseq analysis confirmed that a small number of previously identified miRNAs were differentially expressed in HIV co-infected individuals, major changes in the overall pattern of small RNA subtypes in plasma were not observed. In order to determine the overall effect of HIV infection on miRNA expression, we performed a principal component analysis (PCA) of all the miRNA expression levels. Principal component composite values for each individual did not separate individuals into two clusters based on their HIV status (data not shown). Thus, confirming the absence of a wide alteration of the miRNA expression profile in people co-infected with HIV. This observation raised the possibility of the presence of a confounding factor affecting the miRNA repertoire expression in all the cohorts.

### Oral shedding of KSHV correlates with plasma levels of specific cellular miRNAs

**Fig 7:**
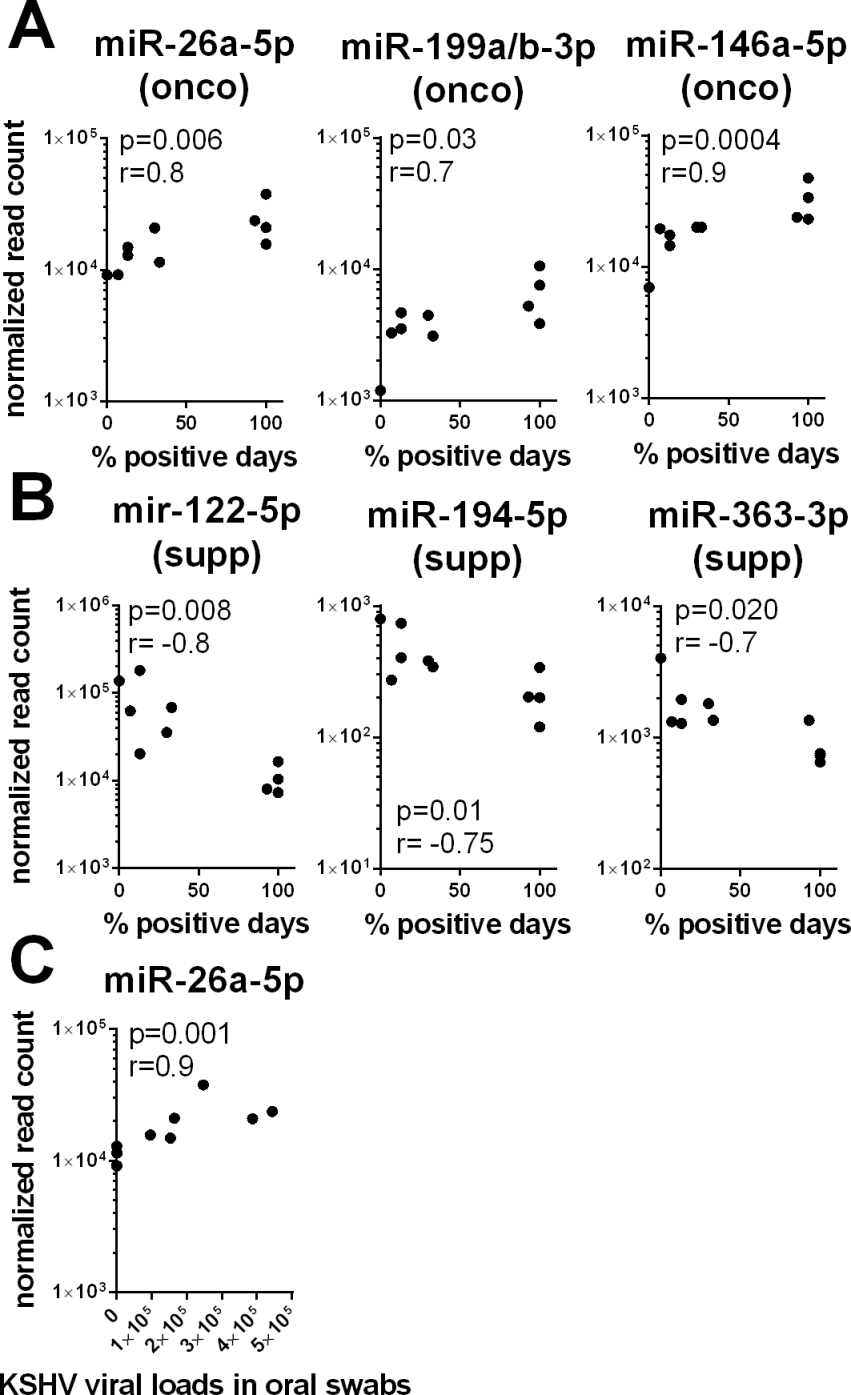
Correlation between miRNA level of expression in plasma and KSHV oral shedding. Spearman correlation scatter plots showing correlations between miRNA normalized read count and KSHV rate of oral shedding (A, B) or KSHV viral loads in oral swab (C) across all the tested individuals are shown. MiRNAs with a statistically significant positive (A) or negative (B) correlation are shown.

Previous studies have utilized the level of KSHV DNA in oropharyngeal swabs as a marker of KSHV replication in oral tissues [40–42]. However, no correlation has yet been established between the risk of malignant disease progression and oral shedding of KSHV. To investigate the possible relationship between KSHV oral shedding and expression of cellular oncomiRs in plasma, we compared the rate of KSHV detection in oral swabs across the 15 day study period with the expression levels of miRNAs in plasma of KSHV-infected individuals with and without HIV co-infection from our RNAseq study. Significant correlations were observed for six cellular miRNAs considered as oncomiRs (Fig 7). The expression levels of miR-26a-5p, miR-146a-5p and miR-199a/b-3p, which are all associated with oncogenic activity, were positively correlated with the rate of KSHV detection (Fig 7A). In contrast, the expression levels of miR-122-5p miR-194-5p, and miR-363-3p, which are all associated with tumor suppressor activity, were negatively correlated with the rate of KSHV detection (Fig 7B).

We also compared the KSHV viral load in the oral swabs with the levels of miRNAs in plasma obtained on the same day. Of the 385 identified cellular miRNAs, only miR-26a-5p had expression levels in plasma that correlated with level of KSHV DNA detected in the oral swab (p = 0.001, r = 0.9) (Fig 7C). No significant correlation was observed between the plasma level of cellular miRNAs and HIV viremia in plasma (data not shown). Thus, our study shows for the first time direct associations between KSHV shedding rate in the oral epithelium and the expression levels of several cellular oncomiRs in plasma.

### Expression of mature KSHV-encoded microRNAs is variable in Ugandan plasma samples

KSHV contains 12 miRNA genes within the latency region of the genome, which encode mature miRNAs from both 5’ (5p) and 3’ (3p) ends of the pre-miRNA stem loop [26, 43, 44]. Since the in vivo expression pattern of these miRNAs has only been described in KSHV-associated malignancies [11, 20, 45], we analyzed our RNAseq data on small RNA in plasma of KSHV-infected individuals without KS (Table 2) for the presence of reads mapping to known KSHV miRNA sequences (miRBase 21) [24]. With the exception of miR-K12-1, reads mapping to either the 5p or 3p sequences from all of the other KSHV miRNAs were detected in at least one individual (Table 3). The miR-K12-8-3p was the most prevalent (7/10) and abundantly expressed KSHV miRNA in plasma from both KSHV+/HIV- and KSHV+/HIV+ groups. Other prevalent KSHV miRNAs included miR-K12-3-3p (6/10), miR-K12-4-3p (5/10), and miR-K12-11-3p (5/10). MiR-K12-4-3p and miR-K12-8-3p have both been shown to be abundantly expressed in KSHV infected PEL lines. Two of the KSHV+/HIV+ individuals, 033–1 and 174–5, showed the highest level of miRNAs in plasma (Table 3). Overall, the expression levels of the KSHV miRNAs were highly variable, with some individuals showing high levels of KSHV miRNAs and others with no detectable miRNAs. Following maturation each 3p-end or 5p-end of the twelve KSHV miRNA stem loop precursors can generate not only a mature miRNA of an expected size but also several length variants called isomiRs. Among the 11 species of KSHV miRNAs detected in the plasma samples, we observed 14 viral miRNAs with a variation in the nucleotide length of their seed sequences (Table 3). Additional experiments are needed to confirm that the most abundant length variants are functional isomiRs and to identify their cellular mRNA targets.

**Table 3:** KSHV associated miRNA in plasma.

		KSHV+/HIV-					KSHV+/HIV+				
		138–9	146–6	169–3	361–0	365–1	033–1	091–1	131–6	174–5	181–8
miRNA	Sequence	Count^c^	Count	Count	Count	Count	Count	Count	Count	Count	Count
miR-K12-2-5p	AACTGTAGTCCGGGTCGAT(CTGA)	1.03	0	0	0	0	9.3	0	0	132.4	0
^a^	ACTGTAGTCCGGGTCGAT(CTG)	0	0	0	0	0	0	0	0	2.04	0
miR-K12-3-3p	TCGCGGTCACAGAATGTGACA	0	0	0	0	0	0	0	0	1.85	0
miR-K12-3-5p	TCACATTCTGAGGACGGCAGCGA(CG)	0.01	2	0.3	0	0	26.3	0	0	217.2	1.8
miR-K12-3_+1_5	ATCACATTCTGAGGACGGCAGCGA	0.02	0	0	1.2	0	4.2	0	0	25.7	0
miR-K12-4-3p	TAGAATACTGAGGCCTAGCTG(AT)	0.01	0	0	0	0	15	0.3	0	186.1	2.2
	AGAATACTGAGGCCTAGCTG(A)	0	0	0	0	0	0	0	0	6.8	0
miR-K12-4-5p	AGCTAAACCGCAGTACTCTAGG	0.04	0	0	0	0	0.8	0	0	23.1	0
miR-K12-5-3p	TAGGATGCCTGGAACTTGCCGG(T)	0	0	0	0	0	1.1	0	0	0	4.8
miR-K12-6-3p	TGATGGTTTTCGGGCTGTTGAG(C)	0	0	0	0	0	4.1	0	0	0	0
miR-K12-7-3p	TGATCCCATGTTGCTGGCGC(TCA)	0	0	0	0	0	3.8	0	0	20.7	0
miR-K12-7-5p	TGAGCGCCACCGGACGGGG(ATT)	0	0	0	0	0	0.3	0	0	7.9	0
miR-K12-8-3p	CTAGGCGCGACTGAGAGAGC(AC)	0.2	1.6	0	0	0.7	64.8	0	1.1	789.2	7.8
	TAGGCGCGACTGAGAGAGC(AC)	0.4	0	0.6	0	0	1	0	0	10.2	0
miR-K12-8-5p	ACTCCCTCACTAACGCCCCGCT	0	0	0	0	0	0	0	0	2.6	1.1
	CTCCCTCACTAACGCCCCGCT	0	0	0	0	0	0	0	0	3.1	0
	CACTCCCTCACTAACGCCCC	0	1.4	0	0	0	0	0	0	0.4	0
	GCACTCCCTCACTAACGCCCC	0	0	0	0	0	0	0	0	3.7	0
miR-K12-9-3p	CTGGGTATACGCAGCTGCGT(AA)	0	0	0	0	0	0.8	0	0	3.5	0
miR-K12-9-5p	ACCCAGCTGCGTAAACCCCG(CT)	0	0	0	0	0	0	0	0	7.9	0
	TACCCAGCTGCGTAAACCCC(G)	0	0	0	0	0	0	0	0	1.3	0
miR-K12-10a	TAGTGTTGTCCCCCCGAGTGG(C)	0	0	0	0	0	3.3	0	0	42.2	0
miR-K12-10b	TGGTGTTGTCCCCCCGAGTGG(C)	0	0	0	0	0	0	0	0	0.4	0
miR-K12-10a_+1_5	TTAGTGTTGTCCCCCCGAGTGG(C)	6	0	0	0	0	0.8	0	0	53.3	0
miR-K12-10b_+1_5	TTGGTGTTGTCCCCCCGAG(TGGC)	0	0	0	0	0	0	0	0	2.4	0
miR-K12-11-3p	TTAATGCTTAGCCTGTGTCCG(AT)	0.02	0	0.9	0	0	8.5	0	0	111.8	10
	TAATGCTTAGCCTGTGTCCGA	0	0	0	0	0	0	0	0	1.1	0
	ATGCTTAGCCTGTGTCCG	0	0	0	0	0	0	0	0	1.8	0
	CCTTAATGCTTAGCCTGTGTCCG	0	0	0	0	0	0	0	0	1.3	0
miR-K12-12-3p	TGGGGGAGGGTGCCCTGGTTG(A)	0	0	0	0	0	0	0	0	7.9	0
	GGGAGGGTGCCCTGGTTGAC	0	0	0	0	0	0	0	0	0.7	0
miR-K12-12-5p	AACCAGGCCACCATTCCTCTCCG	0	0	0	0	0	0	0	0	0	0
	CTAACCAGGCCACCATTCCTCTC	0	0	0	0	0	0	0	0	2.4	0
	TGTCAACCAGGCCACCATT	0	0	0	0	0	0	0	0	2.2	0
Mapped reads^b^		3.9	5.0	3.3	4.8	5.4	6.0	12.8	6.5	5.4	2.7

^a^ The potential isomiR sequences are shown for each KSHV miRNA in the corresponding rows below

^b^Total mapped reads expressed in million reads (*10^6^)

^C^ normalized read count (TPM)

To date, three KSHV miRNAs have been demonstrated to function as mimics of cellular miRNAs. These include KSHV miRNAs, miR-K12-3, miR-K12-10a, and miR-K12-11 which share seed sequence homology with cellular miRNAs, miR-23-3p, miR-142-3p, and miR-155 respectively [46–48]. These three cellular miRNAs have been shown to target pathways involved in cancer or immunity. Therefore, expression of the respective KSHV orthologues of these cellular miRNAs could contribute to the induction of KSHV-associated tumors in infected individuals. Our RNAseq data revealed reads mapping to miR-K12-11 in five individuals. However, we did not detect the miR-K12-11 cellular mimic, miR-155, in plasma from any of the 10 individuals analyzed. In contrast, the cellular mimics for miR-K12-3, miR-K12-10a and miR-K12-10a+1_5 (miR-23a-3p, miR-142-3p, and miR-142-3p-1_5 respectively), were broadly detected in all plasma samples analyzed. Expression of miR-K12-K10a-3p and its isomiR miR-K12-10a+1_5 is of particular interest since it is the only KSHV miRNA with a reported transforming activity [49]. KSHV miR-K12-10a mimics the cellular miRNA, miR-142-3p, targeting several tumor suppressor transcripts. Our results confirm a previous report that miR-K12-10a and miR-K12-10a+1, both derived from the differential processing of the miR-K12-10 stem loop 5’ end, are detected with an equal abundance (individual #174–5) when abundantly expressed [26]. Moreover, miR-K12-10b and miR-K12-10b+1, both derived from an RNA editing event in the seed region of miR-K12-10a, were barely detectable in the same individual, confirming their low level of expression as previously observed for KSHV infected cells. Most importantly, our data showed that KSHV encoded miRNAs with transforming activity can be detected in an extracellular form in plasma from KSHV-infected people with blood viremia.

### KSHV plasma viremia correlates with KSHV-encoded miRNA plasma levels

**Fig 8:**
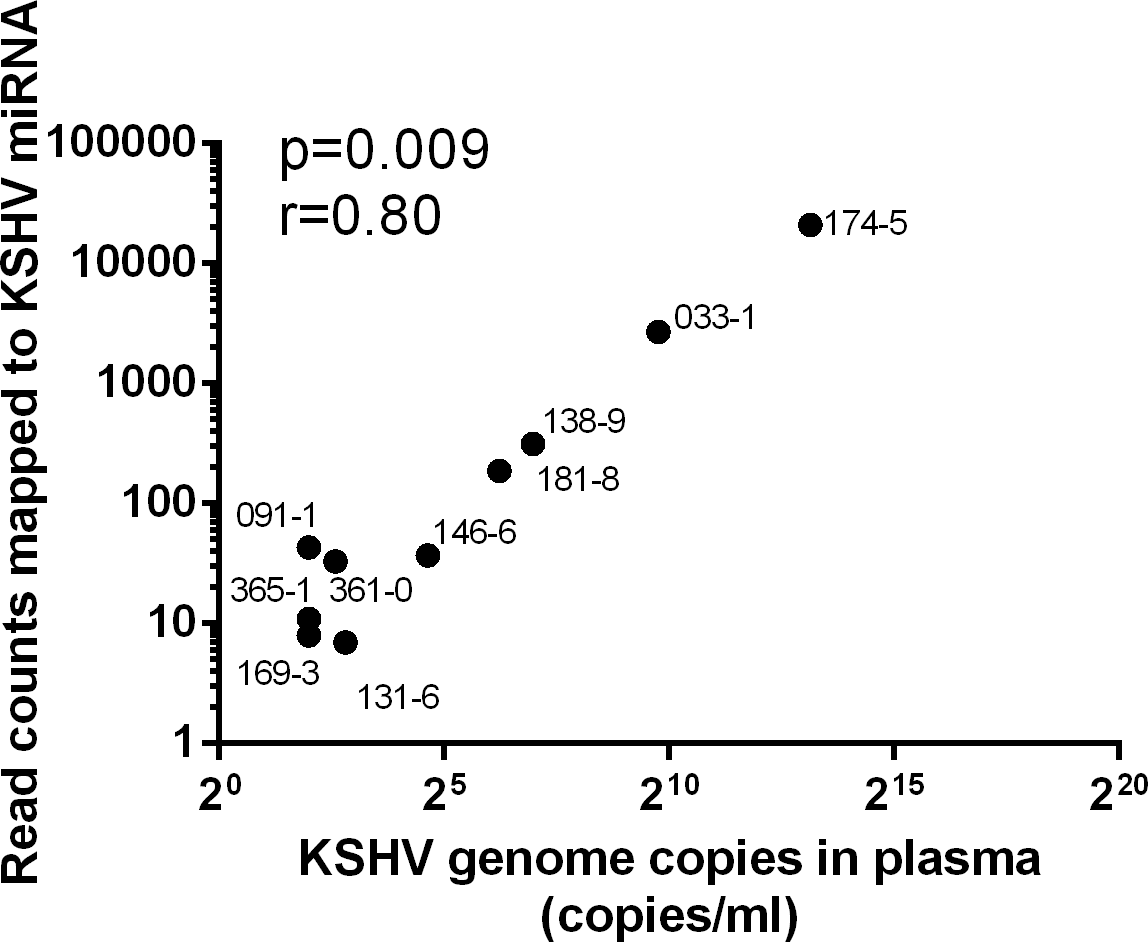
Correlation between KSHV miRNAs expression levels in plasma and KSHV viremia. Spearman correlation scatter plots showing correlations between total read counts for KSHV encoded miRNAs and KSHV plasma viremia across all the tested individuals.

A previous study reported that the viral miRNA cluster encoded by KSHV is actively involved in controlling viral lytic replication [50]. To determine the correlation between KSHV miRNA expression and lytic replication in vivo, we compared the level of extracellular KSHV genomes present in plasma of the 10 KSHV-infected individuals with or without HIV co-infection with the RNAseq read counts mapping to all the known mature KSHV miRNAs. KSHV viremia was determined by quantitating the cell-free KSHV genomes present in the same plasma sample analyzed by RNAseq. Previously published studies have shown that most of KSHV DNA found in plasma is resistant to DNase degradation, indicating that large proportion of the KSHV DNA in plasma is in the form of encapsidated in virions [51]. Furthermore, the levels of KSHV DNA measured in plasma show a correlation with antibody titers to lytic, but not latent, KSHV antigens [52]. These observations suggest that plasma KSHV DNA is mostly associated with KSHV virions and can be considered as a surrogate marker for lytic viral replication. A strong, statistically significant correlation (r = 0.8, p = 0.009) was observed between the level of KSHV genomes and the expression level of mature KSHV miRNAs (Fig 8) in the 10 plasma samples. Thus, the release of both KSHV genomes and mature KSHV-encoded miRNAs into plasma were highly correlated. Furthermore, no correlation was observed between the HIV load in the KSHV+/HIV+ individuals and either the KSHV miRNA expression or viremia in plasma. Recently, it was reported that specific KSHV miRNAs are incorporated into virions [53]. In viremic individuals, viral miRNAs could originate from viral particles present in the blood. However, the miRNAs detected in our plasma samples showed no specific pattern of expression, suggesting that they were not virion-associated. Moreover, four viral miRNAs (miR-k12-3-5p, miR-k12-4-3p, miR-K12-10a and miR-k12-10b) most often found encapsulated in virions were not systematically detected in the plasma of KSHV viremic individuals (Table 3).

Our results provide the first evidence that, under physiological conditions, mature viral miRNAs extracellular expression levels correlate with KSHV plasma viremia. In contrast, expression of KSHV encoded miRNAs was not associated with either KSHV viral loads in oral swabs or KSHV oral shedding rate.

### EBV miRNAs are concurrently expressed with KSHV miRNAs in plasma

EBV associated malignancies like their KSHV counterparts, are also more frequent in HIV infected people [54]. This observation raises the possibility that KSHV and EBV could cooperate in promoting carcinogenesis, and several pleural effusion lymphomas have been reported to be infected with both viruses. Functional evidences supporting cooperation between viral miRNAs encoded by KSHV and EBV have been previously reported [20]. These observations are in agreement with previous studies showing that KSHV-associated pleural effusion lymphomas are more tumorigenic when co-infected by EBV [55, 56]. EBV is known to encode several viral miRNAs and is endemic in Uganda [43]. Thus, we investigated whether circulating EBV miRNAs were present in plasma samples from the KSHV infected individuals. The RNAseq reads obtained from the 10 HIV+/KSHV+ and HIV-/KSHV+ individuals were analyzed for the presence of mature EBV miRNA sequences. As seen in Table 4, ten different EBV miRNA species were detected in the plasma of the KSHV-infected individuals. All of them belong to the EBV BART group of miRNAs. Expression of these viral miRNAs has been reported to confer a significant growth advantage to EBV associated tumors in vivo [57]. The most prevalent miRNA among all individuals independent of HIV status was miR-BART 10. The overall prevalence and total number of reads mapping to EBV miRNAs were higher among people in the KSHV+/HIV+ group. EBV plasma miRNAs also included several potential isomiRs. We identified three potential EBV isomiRs corresponding to length variants of miR-BART8, miR-BART17 and miR-BART19 respectively. Several of the 15 different EBV miRNAs detected, including miR-Bart1-5p, miR-Bart7, miR-Bart19-3p, and miR-Bart17-5p, have been shown to target cellular tumor suppressor transcripts while the activity of others like miR-Bart2-5p, and miR-Bart22, target pathways involved in the immune response [58, 59]. We did not observe a correlation between the levels of EBV and KSHV miRNAs in plasma. However, KSHV and EBV miRNAs were concurrently detected in plasma from 7 out of 10 individuals, confirming that miRNAs encoded by both viruses could cooperate in promoting the development of malignancies.

**Table 4:** EBV associated miRNAs in plasma.

		KSHV+/HIV-					KSHV+/HIV+				
		138–9	146–6	169–3	361–0	365–1	033–1	091–1	131–6	174–5	181–8
miRNA	sequence	Count^b^	Count	Count	Count	Count	Count	Count	Count	Count	Count
miR-BART1-5p	CTTAGTGGAAGTGACGTGCTG(TG)	0	28	0	0	0	14	9	0	0	21
miR-BART2-3p	AGGAGCGATTTGGAGAAAATAAA	0	0	0	0	0	0	0	2	0	0
miR-BART2-5p	TATTTTCTGCATTCGCCCTTG(C)	0	14	0	0	0	0	0	2	0	0
miR-BART6-3p	CGGGGATCGGACTAGCCTTAG(A)	0	7	0	0	1	3	23	0	0	0
miR-BART7	ATCATAGTCCAGTGTCCAGG(GA)	0	0	0	0	0	0	0	7	0	0
miR-BART7-5p	CCTGGACCTTGACTATGAAAC(A)	0	1	0	0	0	0	0	0	0	0
miR-BART8	TACGGTTTCCTAGATTGTACAG(A)	0	0	0	0	0	0	6	0	0	0
miR-BART8-3p	GTCACAATCTATGGGGTCGTAG(A)	0	2	0	0	0	0	4	4	0	0
^a^	TCACAATCTATGGGGTCGTAG	0	0	0	0	0	0	0	3	0	0
miR-BART10	TACATAACCATGGAGTTGGCT(GT)	0	16	0	0	0	23	4	14	3	0
miR-BART13	TGTAACTTGCCAGGGACGGCTGA	0	0	0	0	0	17	0	0	0	0
miR-BART13-5p	ACCGGCTCGTGGCTCGTACAG(A)	0	0	0	0	0	0	0	0	0	3
miR-BART17-5p	TAAGAGGACGCAGGCATACA(AG)	0	0	0	0	0	0	0	0	0	23
	AGAGGACGCAGGCATACA(A)	0	4	0	0	0	17	0	4	0	0
miR-BART19-3p	TTTTGTTTGCTTGGGAATGC(TC)	0	3	0	0	0	17	0	2	0	18
	TGTTTTGTTTGCTTGGGAATG	0	12	0	0	0	1	7	0	0	0
miR-BART22	TTACAAAGTCATGGTCTAGTAGT	0	0	0	0	0	0	3	0	0	0

^a^ The potential isomiR sequences are shown for each KSHV miRNA in the corresponding rows below

^b^ normalized read count (TPM)

### YRNA is the most abundant type of small RNA detected in plasma exosomes of KSHV-infected Ugandans

Current molecular diagnostic assays for KSHV rely on transient and variable detection of KSHV DNA in oral swabs. Exosomes are thought to be continuously released into the blood circulation. In addition, exosomes are enriched in miRNAs [60]. The exosome molecular content, when released from reservoirs of KSHV-infected cells has been shown to include KSHV-encoded miRNAs. As such, exosomal miRNAs could represent an attractive source of biomarkers for the development of new diagnostic assays. Our initial analysis detected a correlation between KSHV plasma viremia and the detection of KSHV-encoded miRNAs in plasma (Fig 8). KSHV miRNAs were barely detectable in plasma of people with low or no detectable viremia, even if the concomitant oral shedding rate was 100% (individual #131–6, Tables 2 and 3). KSHV miRNAs could still be present at a level below our detection limit in the total small RNA fraction isolated from plasma. Extracting small RNAs from exosomes instead of plasma should increase our limit of detection for miRNAs. Therefore, we purified exosomes from the plasma of KSHV-infected individuals to determine whether KSHV miRNAs could be detected in non-viremic individuals testing positive for KSHV DNA in oral swabs.

We used RNAseq to extensively characterize the different small RNAs biotypes including miRNA in purified plasma exosomes isolated from different groups of individuals (Table 5). The plasma samples from two individuals in the KSHV+/HIV+ group (#25–1 and #27–5) were selected for exosome purification, since KSHV oral shedding is more prevalent in people co-infected with HIV. The study participant #025–1 had detectable oral shedding every day during the screening period (100% shedding rate), while # 027–5 had a shedding rate of 7% (Table 5). The cellular miRNA repertoire expression in the plasma from these individuals had been previously assayed (Table 1). For comparison, we selected three KSHV-/HIV- and two KSHV-/HIV+ individuals. This allowed us to examine the presence of KSHV miRNAs in exosomes from individuals that had no detectable KSHV oral shedding during the screening period. Due to the high prevalence of KSHV infection in the Ugandan population, we expected that the majority of these individuals would still be latently infected with KSHV. None of them had detectable KSHV in plasma.

**Table 5:** Patient demographics and viral status.

	KSHV-/HIV-^a^			KSHV-/HIV+		KSHV+/HIV+	
	296–9	300–7	305–2	019–2	023–7	025–1	027–5
Age (year)	23	50	29	38	46	27	41
Gender	F	F	M	F	M	F	F
KSHV in oral swab^**d**^	N.D.^e^	N.D.	N.D.	N.D.	N.D.	1,2*10^6^	N.D.
KSHV rate of shedding^b^	0%	0%	0%	0%	0%	100%	7%
KSHV plasma viremia^**d**^	N.D.	N.D.	N.D.	N.D.	N.D.	N.D.	N.D.
Rate of plasma viremia^**c**^	0%	0%	0%	0%	0%	0%	0%

^a^ KSHV- = consistently tested negative by specific qPCR on oral swab and plasma samples during the screening period

^b^ Rate of oral shedding or rate of plasma viremia calculated as the percentage of qPCR positive days over the 15 days screening session.

^c^ Plasma samples were collected weekly (day0, day7, day15) during the screening session.

^d^ viral load (viral genome copies/ml) in oral swab and plasma collected the same day as the blood sample screened by RNAseq.

^e^ N.D. = below detection level

**Fig 9:**
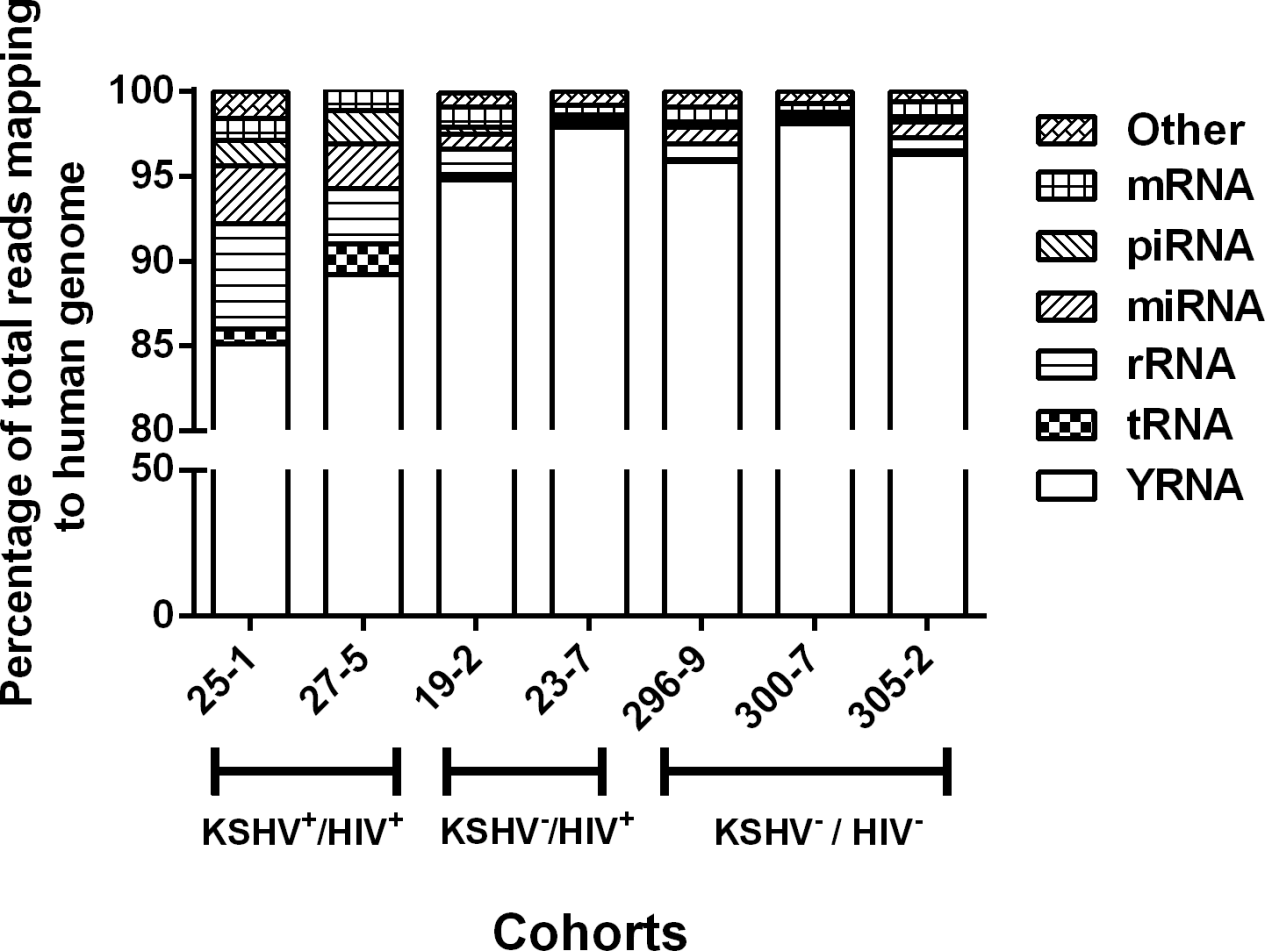
Small RNA biotypes distribution in plasma exosomes. Segments of the bar indicate the percent of reads attributed to each RNA type among all the small RNA reads that mapped to the human reference genome (hg19).

For each plasma, we prepared and sequenced a library of small size exosomal RNA corresponding to an insert size of 11–41 nucleotides (nt). Sequencing reactions for each library produced at least 16 million quality-filtered single-end reads before analysis. The percentage of exosomal reads mapping specifically to ribosomal RNA (rRNA), transfer RNA (tRNA), micro RNA (miRNA), piwi-interacting RNA (piwi-RNA), YRNA and protein-coding RNA (mRNA) are shown in Fig 9. Most small RNA biotypes were detected at a substantially lower percentage than reported in previous studies with an average representation equivalent to 1% or less of the total reads: miRNAs (median 0.9%, range 0.2–3.4), rRNAs (median 0.9% range: 0.2–6.2) and cDNAs (median: 0.9%, range; 0.5–1.3) or less: tRNAs (median 0.2%, range: 0.1–1.8), and piwiRNAs (median: 0.3%, range: 0.1–2.0). The small nucleolar RNAs (snoRNAs) had the lowest distribution, representing only 0.1% or less of the total read count mapping to the human reference genome. In contrast, YRNAs were vastly over-represented, accounting on average for 93% of the total mapped reads (median 95%, range: 85–98). This unusual abundance of YRNA in the plasma exosome fraction was observed in all individuals independently of the KSHV and HIV infection status.

### RNY4 is the most abundant YRNA subtype in plasma exosomes

**Fig 10:**
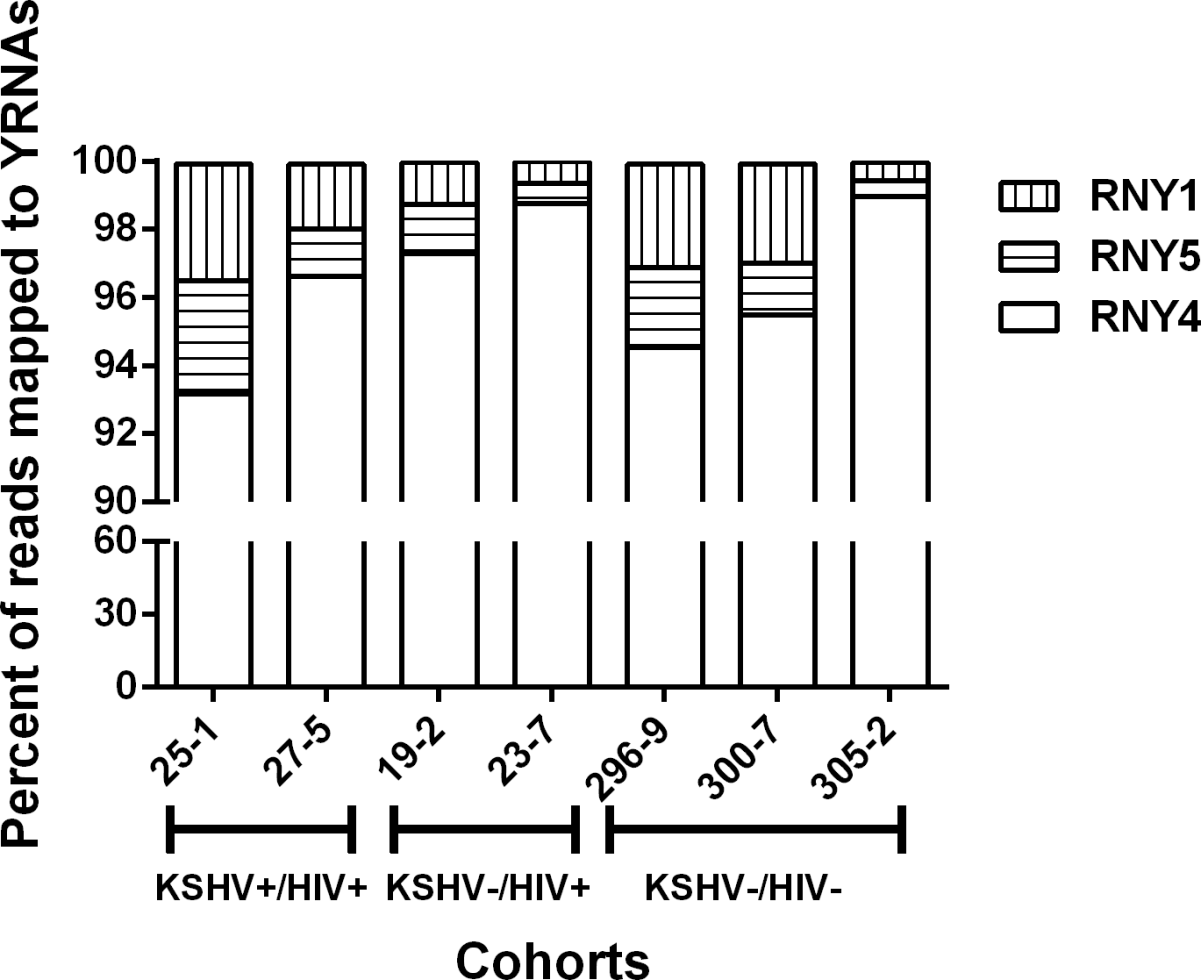
YRNA type distribution in plasma exosomes. Segments of the bar indicate the percent of reads attributed to each YRNA type among all the reads mapping to known YRNA sequences.

YRNAs are small non-coding RNAs that bind to the Ro60 (SSB) and La (SSA) proteins to form ribonucleoprotein complexes. Four different types of YRNA transcripts have previously been described in humans, including RNY1, RNY3, RNY4 and RNY5 [61]. Although the biological function of the YRNAs is still poorly understood, they are thought to be essential factors for chromosomal DNA replication and cell proliferation [62]. To determine the abundance of the different YRNAs, we quantitated the RNAseq reads from the plasma exosome libraries mapping to each type. Only fragments of the YRNAs were detected in our small RNA-seq studies. The vast majority of the fragments were derived from the 5'-ends of specific YRNAs and correspond to the sequences expected from a cleavage within the predicted internal loop [63]. Reads mapping to RNY4 were the most abundant representing an average of 96% of the total YRNA reads (median: 96%, range: 93–98) (Fig 10). This was followed by RNY1 and RNY5 (median 1.8%, range 0.5–3.4 and median 1.4%, range: 0.4–3.3, respectively). The percentage of reads mapping to RNY3 was less than 0.1%. These values are in agreement with a previously published study, which showed that YRNAs are distinctively incorporated in exosomes following a selective mechanism.

### A restricted set of cellular oncomiRs are detected in plasma exosomes

While the vast majority of the sequences in plasma exosome fractions were derived from YRNAs, reads mapping to 273 different cellular miRNAs were also detected. DESEq2 normalized read counts were used to determine the ten most abundant miRNA species in each plasma sample (Table 6). The distribution of the top ten most abundant miRNAs was highly consistent between the different study groups. Nine cellular miRNAs (miR-26a-5p, Let-7a/b/c/d/e/f-5p and miR-10a/b-5p) were systematically present in all samples regardless of the patient viral status. These nine miRNAs are all known oncomiRs. Previous studies have shown that the levels of expression of the Let-7a/b/c/d/e/f-5p miRNAs are altered in KSHV infected cells [64]. In addition, miR-10b-5p is known to be more abundant in exosomes released by KSHV infected cells than within infected cells themselves [45]. Similarly, Let-7a/d/e-5p, and miR-10a have been shown to be preferentially associated with exosomes when released in an extracellular form in plasma, supporting the efficiency of our exosome enrichment procedure [29].

**Table 6:** Most abundant cellular miRNAs detected in plasma exosomes.

KSHV-/HIV-			KSHV-/HIV+		KSHV+/HIV+	
296–9	300–7	305–2	019–2	023–7	025–1	027–5
**miR-26a-5p**^**#**^ (11.1%)*	**miR-26a-5p** (10.9%)	**miR-26a-5p** (10.7%)	**Let-7c-5p** (10.4%)	**Let-7c-5p** (9.5%)	**Let-7c-5p** (9.7%)	**miR-26a-5p** (11.4%)
**Let-7a-5** (8.8%)	**Let-7a-5p** (9.7%)	**Let-7c-5p** (9.3%)	**miR-26a-5p** (7.5%)	**miR-26a-5p** (8.7%)	**miR-26a-5p** (9.2%)	**Let-7a-5p** (9.6%)
**Let-7c-5p** (8.6%)	**Let-7f-5p** (9.1%)	**Let-7a-5p** (9.3%)	**Let-7a-5p** (7.5%)	**Let-7a-5p** (8.2%)	**Let-7a-5p** (8.9%)	**Let-7c-5p** (9.1%)
**Let-7f-5p** (8.2%)	**Let-7c-5p** (9.0%)	**Let-7f-5p** (8.6%)	**Let-7b-5p** (7.4%)	**Let-7f-5p** (7.4%)	**Let-7f-5p** (8.0%)	**Let-7f-5p** (8.8%)
**Let-7b-5p** (5.1%)	**Let-7b-5p** (5.1%)	**Let-7b-5p** (5.4%)	**Let-7f-5p** (6.5%)	**Let-7b-5p** (5.9%)	**Let-7b-5p** (5.7%)	**Let-7e-5p** (5.0%)
**Let-7e-5p** (4.1%)	**Let-7e-5p** (4.6%)	**Let-7e-5p** (4.6%)	**miR-10b-5p** (4.8%)	**let-7e-5p** (4.3%)	**miR-10b-5p** (5.6%)	**Let-7b-5p** (4.8%)
**Let-7d-5p** (3.9%)	**Let-7d-5p** (4.2%)	**Let-7d-5p** (4.3%)	**miR-10a-5p** (4.7%)	**miR-10b-5p** (4.2%)	**miR-10a-5p** (5.5%)	**Let-7d-5p** (4.5%)
Let-7i-5p (3.8%)	Let-7i-5p (3.7%)	**miR-10b-5p** (3.5%)	**Let-7e-5p** (4.0%)	**miR-10b-5p** (4.1%)	**Let-7e-5p** (4.7%)	Let-7i-5p (3%)
**miR-10b-5p** (3.3%)	**miR-10b-5p** (2.9%)	**miR-10a-5p** (3.5%)	**Let-7d-5p** (3.6%)	**let-7d-5p** (3.7%)	**Let-7d-5p** (3.8%)	**miR-10b-5p** (2.9%)
**miR-10a-5p** (3.2%)	**miR-10a-5p** (2.8%)	Let-7i-5p (3.4%)	Let-7i-5p (2.4%)	Let-7i-5p (2.5%)	miR-122-5p (2.2%)	**miR-10a-5p** (2.9%)

#miRNAs shared between all the patients are shown in bold

*Percentage of each miRNA related to the total mapped cellular miRNA reads based on Normalized read counts (DEseq2) shown in parenthesis

Only one KSHV miRNA, miR-K12-3 (study participant # 027–5, KSHV+//HIV+) and two EBV miRNAs, miR-BART17-5p and miR-BART22 (individuals identified as #025–1, KSHV+/HIV+ and #019–2, KSHV-/HIV+, respectively) were detected in the exosomes (Table 7).

**Table 7:** Viral miRNAs detected in plasma exosomes.

		KSHV+/HIV+		KSHV-/HIV+		HIV-/KSHV-		
		025–1	027–5	019–2	023–7	296–9	300–1	305–2
**miRNA**	Sequence	count	count	count	count	count	count	count
**miR-K12-3-5p**^**a**^	ATCACATTCTGAGGACGGCAGCGA	0	17	0	0	0	0	0
**miR-BART17-5p**	TAAGAGGACGCAGGCATACAAG	4	0	0	0	0	0	0
**miR-BART22**^**B**^	TACAAAGTCATGGTCTAGTAGT	0	0	7	0	0	0	0

^**A**^ potential isomiR form of miR-K12-3-5p

^**B**^ potential isomiR form of miR-BART22

Thus, the vast over-representation of YRNAs in the exosomal small RNA fraction severely impaired our ability to detect viral miRNAs as a diagnostic assay for KSHV infection. The consistently high level of exosomal YRNAs in our cohort of individuals from Uganda suggests the presence of a confounding factor affecting these individuals independent of KSHV or HIV status.

### The proteomic profile of the plasma exosomes is compatible with erythrocyte or keratinocyte origins

Previous studies have shown that the human erythrocyte transcriptome shows a significant enrichment in YRNA transcripts, including the RNY1 and RNY4 types [65, 66]. Erythrocytes are also known to release exosome-like microvesicles in response to infection with the plasmodium parasite responsible for malaria [67]. Since malaria, like KSHV and HIV, is endemic in Uganda, the over-representation of YRNAs in plasma exosomes could be due to microvesicles released by erythrocytes in response to plasmodium infection. Since exosomes are known to contain protein constituents that are markers of the specific cell type releasing the exosomes, we characterized the proteomic profile in exosomes isolated from Ugandan plasma samples using quantitative mass spectrometry. We used Tandem Mass Tag (TMT) since it provides data identifying proteins and comparing their relative abundances in multiple samples concurrently.

**Fig 11:**
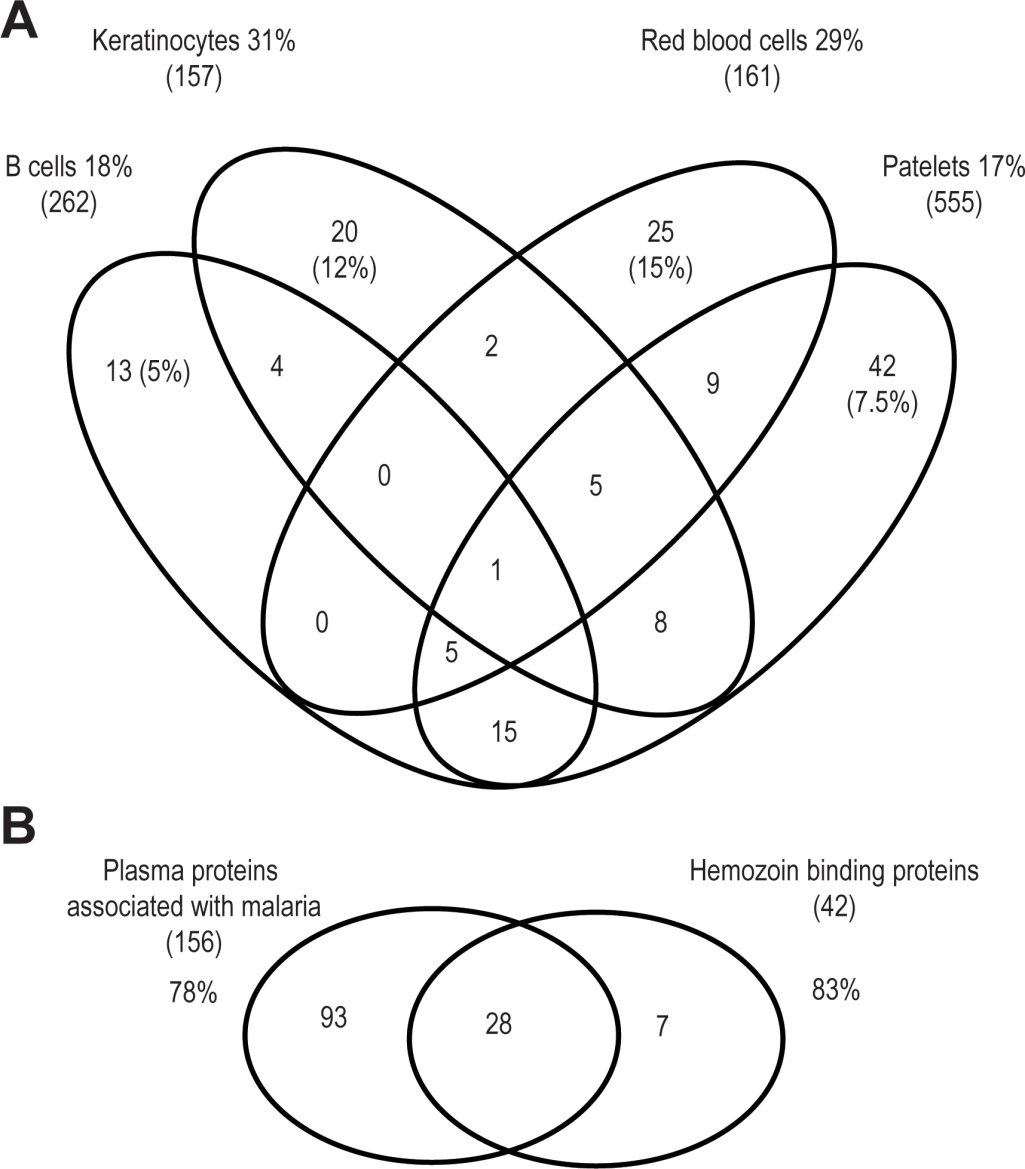
Proteomic profile of Ugandan plasma exosomes. Venn diagram of the distribution of plasma exosomal proteins that are associated with extracellular vesicles released from different cell types (A) or with cellular proteins interacting with hemozoin (B).

We analyzed the proteome of the exosomal fraction of plasma from KSHV infected individuals that were either HIV+ or HIV-, using the same two cohorts as previously described in Table 2. Due to insufficient plasma samples from two individuals of the original HIV infected group (131–6 and 181–8), plasma from two other KSHV+/HIV+ individuals (#025–1 and #037–2) were used for this analysis. Plasma exosomes were purified from all ten individuals both in the KSHV+/HIV+ and the KSHV+/HIV- groups, and the total proteins were extracted and processed for mass spectrometry analysis. Mass spectrometry analysis identified a total of 1676 peptides from 419 different proteins in the exosomal fractions of the plasma samples. No peptides derived from either KSHV or EBV viral proteins were detected. The panel of exosomal proteins was compared to several databases of proteins associated with exosomes and extracellular vesicles isolated from different cell types [68]. As seen in the Venn diagram in Fig 11A, 31% (49/157) of the proteins commonly detected in extracellular vesicles released by keratinocytes were identified in our panel of exosomal proteins. Similarly, 29% (47/161) of the proteins commonly detected in extracellular erythrocyte vesicles were also detected in the exosomal proteome. The similarity was smaller with proteins from vesicles released from platelets (94/555, 17%) and B cells (47/262, 18%). The exosomal proteins from different types of endothelial cells showed overlaps of 10% or less (data not shown). Comparison with proteins uniquely associated with extracellular vesicles released from specific cell types showed the highest similarity with proteins from erythrocytes (25/161; 15%), followed by keratinocytes (20/157; 12%), platelets (42/555; 7.5%) and B cells (13/262; 5%) (Fig 11A).

### The proteomic content of plasma exosomes matches the plasma proteome of individuals infected with *Plasmodium falciparum*

Previous mass spectrometry analysis of plasma samples from malaria patients and healthy individuals revealed differential expression for several host plasma proteins including proteins known for their ability to bind the malarial pigment hemozoin [69]. During the intraerythrocytic stage, the plasmodium parasite digests hemoglobin resulting in production and release of hemozoin. Due to its amphipathic nature, hemozoin binds to several plasma proteins. Proteins interacting with hemozoin represent a specific protein profile signature that could be useful for malaria diagnostic. Therefore, we determined the number of these specific host proteins present in the plasma exosome proteomes from individuals in both the KSHV+/HIV+ and KSHV+/HIV- groups to determine possible malaria exposure. As seen in Fig 11B, 78% (121/156) of the plasma proteins associated with malaria infection were detected in the exosomal proteomes of the Ugandan study participants. Furthermore, 83% (35/42) of the cellular proteins known to interact with hemozoin were also detected in the plasma exosomes. These similarities were observed in each of the plasma exosome samples, suggesting that the KSHV-infected Ugandans in our study were co-infected with *Plasmodium falciparum*.

### The proteomic content of the plasma exosome from Ugandan individuals includes *Plasmodium falciparum* proteins

To unequivocally determine the malaria infection status of individuals in the KSHV+/HIV+ and KSHV+/HIV- groups, we searched the mass spectrometry data sets for matching peptides with a *Plasmodium falciparum* protein database (reference strain 3D7). This analysis identified a set of 11 plasmodium proteins in the plasma exosomes of every Ugandan sample. Using the Proteome Discoverer software (ThermoFisher) we determined the relative abundance of each plasmodium peptide detected in the plasma exosomes of KSHV-infected individuals with or without HIV co-infection (Table 8).

**Table 8:** Relative abundance of plasmodium falciparum peptides in exosomal protein lysates.

	KSHV+/HIV-					KSHV+/HIV+				
	138–9	146–6	169–3	361–0	365–1	025–1	033–1	037–2	091–1	174–5
PF3D7_1460800	51^**a**^	74	187	56	48	77	150	121	70	162
PF3D7_1239800	30	55	180	57	75	67	184	112	69	168
PF3D7_0914900	109	23	146	22	205	23	59	231	49	129
PF3D7_0802700	127	109	145	152	106	121	33	66	79	58
PF3D7_0313900	106	75	94	91	173	110	116	84	76	71
PF3D7_0410800	85	115	86	93	81	96	130	92	82	136
PF3D7_0823500	73	103	79	80	96	89	128	132	98	118
PF3D7_0831700	61	55	75	45	96	79	78	94	331	83
PF3D7_0522400	74	101	59	200	121	96	66	49	112	119
PF3D7_1002700	81	110	50	110	99	161	65	96	160	63
PF3D7_1014600	207	115	48	70	127	50	78	49	75	177

^a^ Relative peptide abundance calculate using the Proteome Discoverer software.

Plasma samples were all tested negative using a commercial malaria rapid diagnostic test targeting the HRP-2 and aldolase (Bionaxnow malaria, Alere) suggesting a low level of parasitemia compatible with an asymptomatic malaria infection stage (data not shown). Moreover, subsequent analyses of the initial RNAseq data revealed the presence of a substantial number of RNA reads mapping specifically to the plasmodium falciparum reference genome sequence (strain 3D7) in all individuals from both groups.

These observations confirm the presence ongoing *Plasmodium falciparum* infections in study participants from different cohorts.

### *Plasmodium falciparum* proteins in plasma exosomes are potential biomarkers of malaria infection

**Fig 12:**
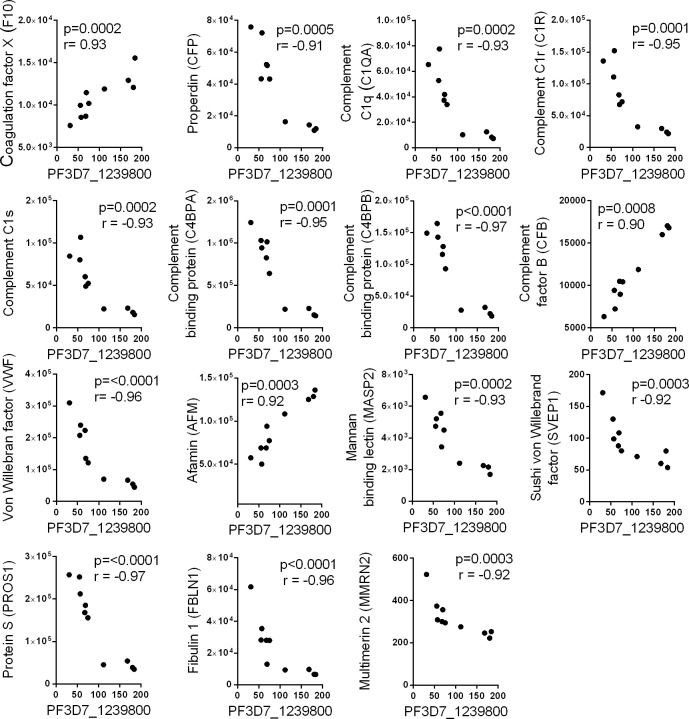
Correlation between the relative abundance of the malarial protein PF3D7_1239800 and cellular proteins in exosomes. Spearman correlation scatter plots showing strong correlations between the relative abundance of peptide from the malarial protein PF3D7_1239800 and peptides derived from different cellular proteins.

**Fig 13:**
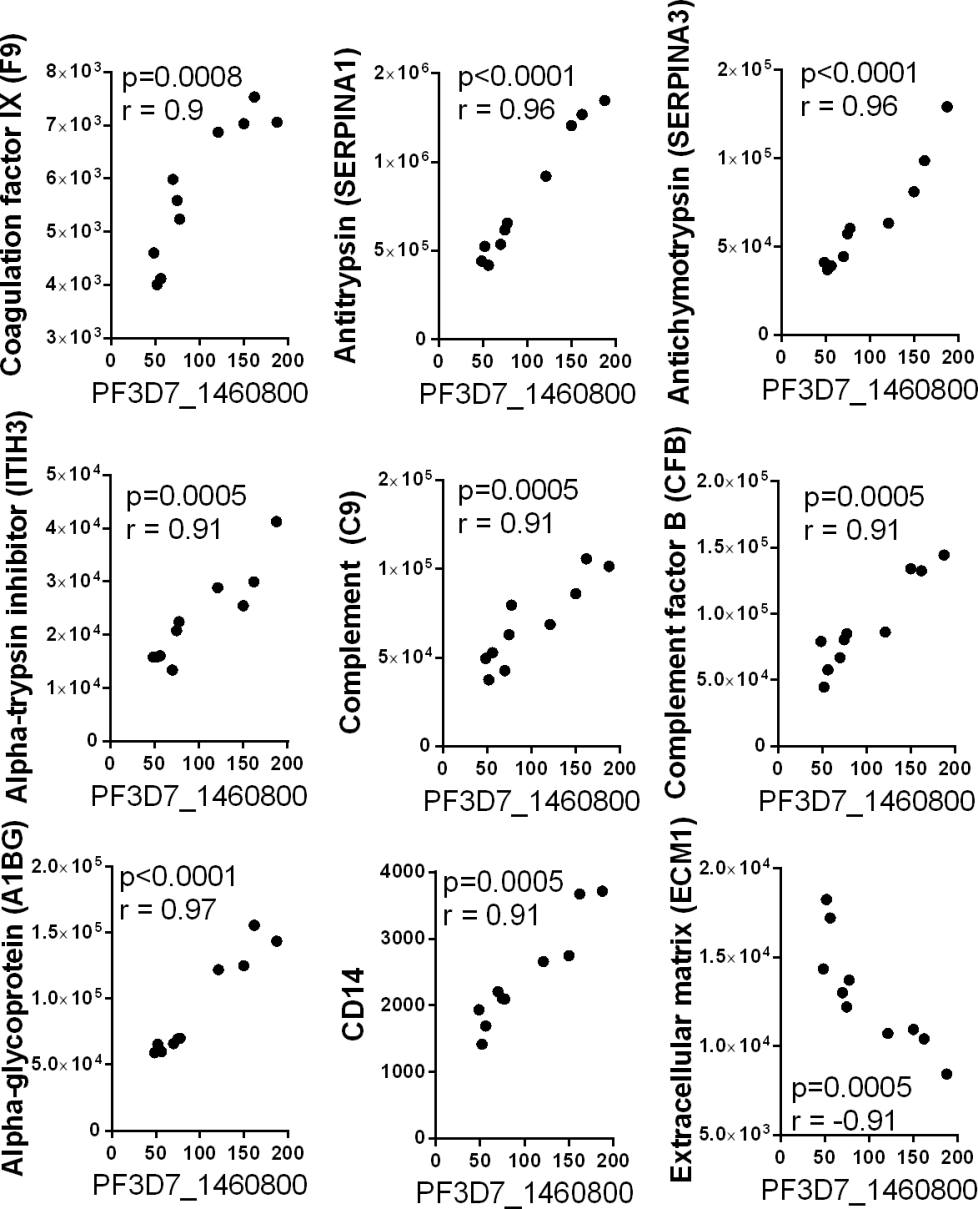
Correlation between the relative abundance of the malarial protein PF3D7_1460800 and cellular proteins in exosomes. Spearman correlation scatter plots showing strong correlations between the relative abundance of peptide from the malarial protein PF3D7_1460800 and peptides derived from different cellular proteins.

**Fig 14:**
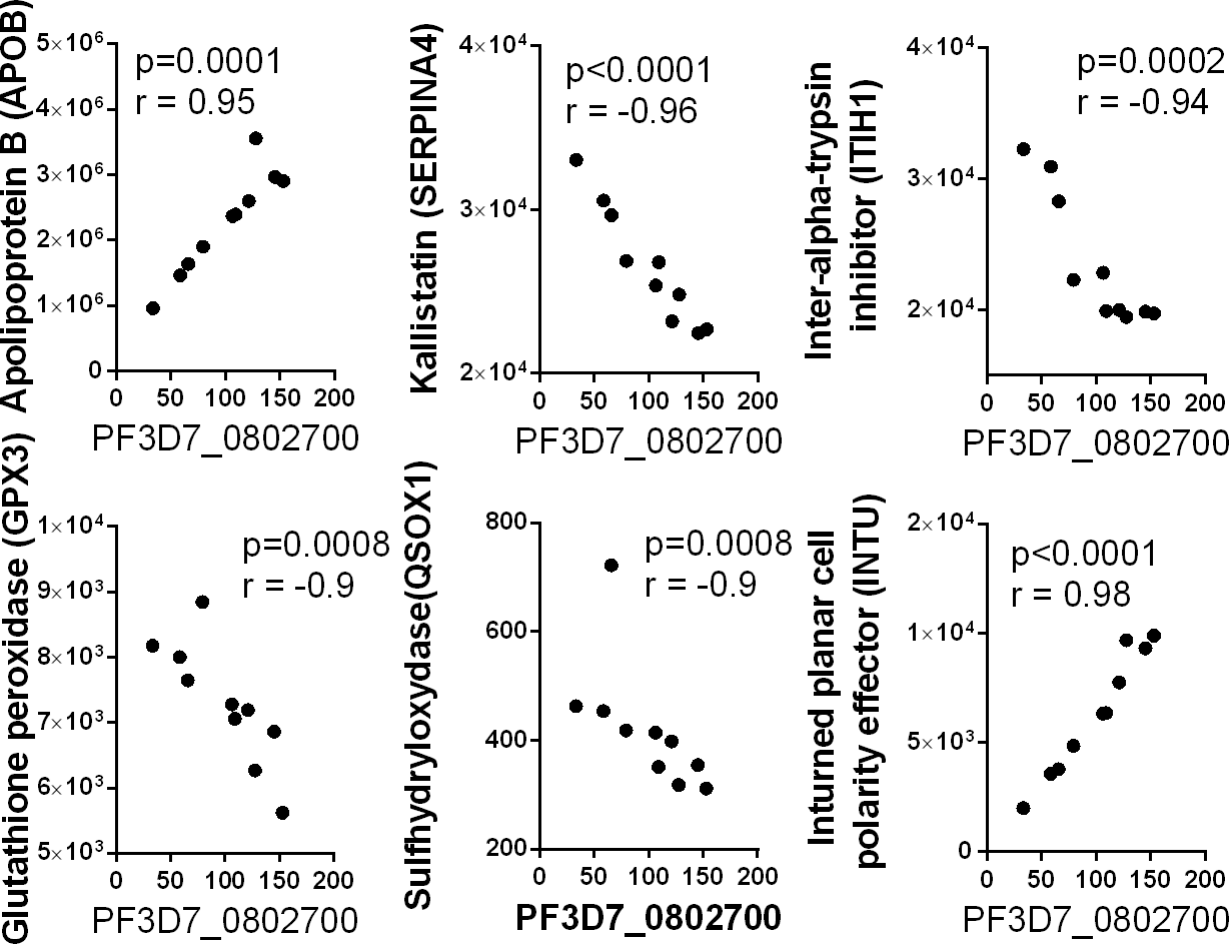
Correlation between the relative abundance of the malarial protein PF3D7_0802700 and cellular proteins in plasma exosomes. Spearman correlation scatter plots showing strong correlations between the relative abundance of peptide from the malarial protein PF3D7_0802700 and peptides derived from different cellular proteins.

Several studies have used proteomic approaches to identify new plasma biomarkers for diagnosis of malaria infections [70–72]. In order to evaluate the biological potential of the exosomal plasmodium proteins as biomarkers, we compared to the relative abundance of peptides derived from plasmodium and cellular proteins in corresponding exosome samples. Malaria protein PF3D7_1239800, of unknown function, was significantly correlated with fifteen of the 419 cellular proteins detected in the 10 plasma exosome samples (Fig 12). These proteins are involved in multiple biological processes including complement cascade, oxidative stress, coagulation and inflammatory response. Positive correlations were observed with the coagulation factor X (F10), complement factor B (CFB) and Afamin (AFM). Negative correlations were observed with a number of cellular proteins, including complement-associated proteins C1QA, C1R, C1S, C4BPA, C4BPB, Von Willebran factor (VWF), properdin (CFP), Mannan-binding lectin serine peptidase 2 (MASP2), and Fibulin1 (FBLN1). Interestingly, a large number of these proteins were previously reported as biomarkers for malaria infection, as the levels of these proteins were significantly altered in the plasma of patients with malaria. Other cellular plasma protein including Multimerin 2 (MMRN2), and protein S (PROS1), are also involved in coagulation processes, but they have not previously been reported as associated with malaria infection. Significant correlations were observed between malaria protein PF3D7_1460800 and the relative abundance of nine cellular proteins (Fig 13). The extracellular matrix protein 1 showed a negative correlation, while the correlations with other eight proteins, including complement protein (C9,) alpha-antitrypsin proteins (SERPINA1, SERPINA3) and the coagulation factor IX (F9) were all positive. Other cellular proteins correlating with PF3D7_1460800 included alpha-1-B glycoprotein (A1BG), complement factor B (CFB), and CD14, which were previously reported to be differentially expressed in malaria patients [73–75]. Finally, the plasmodium protein PH3D7_0802700 showed strong correlations with six cellular proteins, including apolipoprotein (APOB) and kallistatin (SERPINA4) (Fig 14). The APOB plasma protein has been previously reported to have an altered expression profile in patients with malaria [70].

The three *Plasmodium falciparum* proteins, PH3D7_1239800, PH3D7_1460800, and PH3D7_0802700 detected in the plasma exosomes of Ugandan individuals have been poorly studied, and their respective functions are still unknown. However, the strong correlations between the relative abundance of peptides derived from these proteins in plasma exosomes and cellular proteins associated with malaria infections indicates that these peptides could constitute new biomarkers for the diagnosis of either asymptomatic or uncomplicated *Plasmodium falciparum* infections.

## Conclusions

A recent study examined the miRNA repertoire expression in individuals with KSHV associated malignancies [11]. Extracellular KSHV encoded miRNAs and host oncomiRs were readily detected in plasma and pleural fluid suggesting that the viral and cellular miRNAs could be novel biomarkers for disease progression. MiRNAs can associate with lipoproteins and extracellular vesicles, which renders them remarkably stable in plasma and allows them to be transferred between cells and perform regulatory activity in distant cells. These observations support a model in which extracellular viral and host miRNAs could trigger activation of signaling pathways in a paracrine manner, promoting KSHV pathogenesis in patients with existing malignant tumors. In the current study, we provide evidence that the repertoire of extracellular miRNAs circulating in plasma is altered in KSHV-infected individuals prior to the development of KSHV-associated malignancies, suggesting a role for KSHV-induced miRNAs in the initial induction of disease as well as tumor progression. Furthermore, our study reveales that HIV co-infection induced additional changes in the miRNA repertoire that could further promote an environment conducive to tumor development.

Our study was based in Uganda, a country where KSHV is endemic. We compared the extracellular miRNA repertoire in individuals with or without detectable KSHV oral shedding during the study period. Using the Nanostring platform to detect and quantify mature human miRNAs, we observed that the expression levels of several circulating oncomiRs were significantly altered in plasma from individuals with detectable oral shedding of KSHV during the study period. We observed a systematic increase in oncogenic miRNAs and decrease in tumor suppressor miRNAs compared to a group of people without detectable oral shedding. Among the 26 differently expressed mature miRNAs, Let-7i-5p, miR-21-5p, miR-93-5p, miR-106a—5p, miR-142-3p, miR-146a-5p, and miR-221-3p were previously reported to be altered in KSHV infected cell cultures *in vitro* [19]. This suggests that changes in the miRNA repertoire observed in plasma would reflect alterations in miRNA expression in KSHV-infected cells in tissues including the oral epithelium. Overall, our observations support a model of paracrine oncogenesis for KSHV in which altered expression and secretion of oncomiRs in infected individuals precede the development of KS lesions and thereby play an important role in promoting induction and progression of disease. Extracellular oncogenic and tumor suppressor miRNAs released by KSHV infected cells could mediate specific regulatory effects on gene transcription in distant cells. While individuals in the control group did not have detectable oral shedding of KSHV during the screening period, it is likely that the majority were latently infected without overt shedding or viremia. Thus, alterations in oncomiR expression could be due to an activated stage of KSHV infection in the oropharyngeal cavity associated with increased oral shedding of KSHV.

Since HIV is also endemic in Uganda, we characterized the miRNA repertoire expression in plasma collected from individuals dually infected with KSHV and HIV before and after ART treatment. Individuals co-infected with HIV and KSHV showed similar alterations of the oncomiR repertoire expression to that seen in KSHV-infected individuals, but often these changes were exacerbated in the context of HIV. These alterations would result in a more favorable environment for the development of KS or other KSHV-associated malignancies in immunosuppressed patients. Interestingly, the expression levels of several oncogenic miRNAs including miR-93-5p, miR-130a-3p, miR-199a/b-3p, mir-223-3p, and miR-361-5p were significant decreased after two years of anti-HIV drug treatment consisting of a combination of nucleotide, nucleoside, and non-nucleoside reverse transcriptase inhibitors (lamivudine, tenofovir and efavirenz). Over the past years, evidence has accumulated that ART therapy results in a substantial decrease in the incidence of KS in KSHV+/HIV+ co-infected individuals [76]. This effect has been mostly attributed to restoration of the immune response affected by HIV, leading to a regain in immune control of the KSHV infection. In addition, we have previously shown that anti-retroviral drug regimens that include protease inhibitors such as nelfinavir, are associated with a lower frequency of KSHV oral shedding among individuals co-infected with KSHV and HIV [77]. However, a decrease in extracellular oncogenic miRNA expression levels was observed following 2 years of ART treatment that does not include a protease inhibitor. Our study suggests that tumorigenic miRNAs whose expression is altered by ART treatment are potential biomarkers for monitoring patient’s response to ART treatment and ultimately the risk of progression to KS.

Using high throughput sequencing (RNAseq) we characterized the repertoire of small RNAs in plasmas of KSHV-infected and KSHV/HIV co-infected individuals. The relative representation of each small RNA type was similar between the two groups with the miRNA constituting the most abundant type of small RNA. Differential expression analysis using the DESeq2 software confirmed our initial Nanostring observations that the plasma expression levels of several miRNAs were significantly altered in individuals co-infected with KSHV and HIV. Both analyses concurred on a decrease in the level of the tumor suppressor miR-451a and an increase in the levels of the oncogenic miR-221-3p, miR-199a-5p, and miR-199a/b-3p. The increased expression of miR-221-3p was noteworthy since it is considered to be an essential regulator of angiogenesis, a hallmark of KS [18]. A previous study provided evidence that miR-221-3p plays a role in coordinating endothelial tip cell proliferation and migration [78]. MiR-221-3p regulates endothelial cell proliferation by targeting the cellular transcript for cdkn1b, a known inhibitor of transformation [79]. Cdkn1b is also recognized as a cellular target of KSHV encoded miR-K12-K10a and miR-K12-K10a+1 [49]. The transforming activity of these viral miRNAs could be due to downregulation of cdkn1b resulting in a bypass of the cell cycle arrest checkpoint and an increase in cell proliferation.

In a previous study, we determined that the quantity of KSHV DNA detected on oral swabs was highly correlated with the rate of oral shedding over time [40]. However, the correlation between oral shedding and the clinical aspects of KSHV infection and disease progression is poorly understood. In the current study, we observed for the first time a correlation between KSHV oral shedding rate and the expression levels of six different mature human miRNAs. The levels of the oncogenic miR-26a-5p, miR-146a-5p and miR-199a/b-3p, showed a positive correlation with the oral shedding rate, while the levels of the tumor suppressors, miR-194-5p, miR-122-5p and miR-363-3p showed a negative correlation with the oral shedding rate. Our observations suggest that an increase in oral shedding among immuno-compromised patients could be an important factor for disease progression by altering the extracellular miRNA repertoire expression. Interestingly, the six miRNAs correlated with oral shedding have in common the targeting of the TGF-b signaling pathway. MiR-194-5p [80], miR-199a/b-3p [81, 82], miR-26a-5p [83], and miR-146a-5p [84] regulate the expression of different proteins involved in the canonical TGF-b signaling pathway (or smad pathway) while miR-122-5p targets proteins involved in the non-canonical TGF-b pathway (non-smad pathway) [85]. The remaining miRNA, miR363-3p, is a member of the miR17/92 cluster which is up-regulated in KSHV latently infected cells by vFLIP and vCyclin and can also suppress the TGF-b signaling pathway [86]. The TGF-b pathway is a tumor suppressor pathway regulating many important cellular processes including proliferation, differentiation, survival, and adhesion [87]. Inhibition of the TGF-b pathway is recognized as a critical step in the early stages of carcinogenesis [88]. The importance of the TGF-b pathway for KSHV infection is reflected by the number of different mechanisms KSHV has developed to interfere with this signaling pathway, including inhibition by the viral proteins ORF73 [89], K8 (K-bzip) [90] or by intracellular KSHV-encoded miRNAs in addition to host miRNAs induced by KSHV [86].

Our analysis showed a strong correlation in all KSHV-infected individuals between the level of expression of miR-26a-5p in plasma and the quantity of KSHV DNA detected in oral swabs. Interestingly, miR-26a has been shown to strongly suppress expression of both signal transducer and activator of transcription 3 (STAT3) and the interleukin-6 (Il-6) [91, 92]. In contrast, KSHV infection has been reported to induce STAT3 activation and IL-6 secretion [93, 94]. This suggests that the variable level of miR-26a in plasma could be part of a negative feedback mechanism in response to KSHV activation that would suppress STAT3 activation and IL-6 secretion. Such a regulatory mechanism could lead to the intermittent oral shedding pattern observed in KSHV-infected individuals. Overall our data suggests that episodes of KSHV reactivation in the oral epithelium are associated with a concomitant increase in the expression of host miRNA targeting different signaling pathways including the TGF-b and IL-6/JAK/STAT pathways. Importantly, this paracrine process could promote cell survival and contribute to malignant cellular transformation

Several reports have shown that KSHV viral miRNAs can support tumorigenic processes by perturbing pathways such as apoptosis and immune evasion, or promoting angiogenesis [95–97]. Furthermore, KSHV miRNAs have been detected by PCR in pleural fluid of individuals with KSHV-associated malignancies [11]. We show for the first time the presence of mature KSHV encoded miRNAs in plasma from infected individuals with no clinical signs of associated malignancies. Our study provides the first description of the KSHV encoded miRNA repertoire expressed in plasma in the context of a natural KSHV infection prior to the development of KSHV associated malignancies. KSHV miRNAs were not systematically detected in plasma of infected individuals without KS but were variably detected in individuals within the different study groups. We observed a strong positive correlation between the levels of extracellular KSHV miRNAs in plasma and the number of KSHV genome copies in plasma. Our observations suggest that KSHV plasma viremia, likely resulting from viral replication, is associated with the release of viral miRNAs into plasma. The release of KSHV-encoded miRNAs in plasma in conjunction with the release of cellular oncomiRs could promote a micro-environment favorable for KSHV carcinogenesis. The most abundant KSHV miRNAs detected in plasma from viremic individuals included miR-K12-8-3p and miR-K12-3-5p, miR-K12-4-3p, and miR-K12-11-3p. These viral miRNAs have in common targeting the TGF-b pathway. Both miR-K12-8-3p and miR-K12-3-5p induce a strong downregulation of TGF-b [98], while miR-K12-4-3p and miR-K12-11-3p target GRB2 and SMAD5 [99, 100] respectively, also contributing to down-modulation of the TGF-b mediated signal transduction. To our knowledge, miR-K12-10a-3p and its nucleotide variant, miR-K12-10a+1-3p, are the only two KSHV miRNAs to have a transforming activity [49]. In addition, miR-K12-10a and its associated variant also target cellular proteins from the TGF-b signaling pathway. The detection of extracellular miR-K12-10a and miR-K12-10a+1_3p miRNAs in plasma of KSHV-infected individuals suggests that the transforming activity of these viral miRNAs may not be limited to KSHV-infected cells but could extend to distant uninfected cells.

KSHV and EBV are both oncogenic lymphotropic viruses and both encode several viral miRNAs. Interestingly, miRNAs from both viruses target several similar cellular signaling pathways, including TGF-b. This suggests that in co-infected cells, miRNAs from both viruses could synergize in promoting pathogenesis. This hypothesis is supported by the fact that KSHV-associated pleural effusion lymphomas are more tumorigenic when co-infected by EBV [55, 56]. Our study showed that in several individuals, both EBV and KSHV miRNAs are concomitantly detected in plasma. This suggests that the opportunity for cooperation between viral miRNAs from both viruses to promote cell transformation is not limited to co-infected cells but could extend to distant cells.

We also characterized the small RNAs present in exosomes purified from plasma of our different Ugandan cohorts. Paradoxically, we observed a discrepancy between the relative representations of two different types of small RNA compared to previously published observations. Typically, small RNAs of the miRNA type are selectively enriched in plasma exosomes and can represent up to 70% of the total exosomal small RNAs. Another type of small RNA, the YRNAs, is also actively exported in exosomes, and generally accounts for approximately 14% of the total exosomal small RNAs. The Ugandan cohorts in our study, miRNAs represented on average 41% of the total small RNAs in plasma but only 1% of the small RNAs in plasma exosomes. In contrast, YRNAs represented on average for 5% of the total small RNAs in plasma, but as much as 93% of the exosomal small RNAs detected in our cohorts. Small RNAs present in extracellular fluids, such as plasma, are protected from RNases through packaging in extracellular vesicles, such as exosomes and lipoproteins. In contrast with the procedure used for total RNA isolation in plasma, the methods used in our study to isolate exosomes (exoquick-LP and Qiagen exoRNeasy) included specific steps to reduce lipoprotein particles contamination. The exoquick-LP protocol allows for preclearance of lipoprotein particles using proprietary reagents while the Qiagen exoRNeasy uses membrane affinity columns to specifically isolate exosomes. Therefore, the miRNAs in our plasma exosome fractions would be mostly depleted from those associated with LDL or HDL lipoproteins. Our analysis revealed that the miRNA repertoire in the plasma exosomes was different than the repertoire in the total plasma confirming a selective enrichment of specific miRNAs in exosomes. Our data suggests that in our Ugandan cohorts, the majority of extracellular miRNAs in plasma are associated with vesicles other than exosomes, most probably with either high or low-density lipoprotein (HDL or LDL). Lipoproteins are the most abundant lipid particles in plasma and serum. Similarly to what has been described for miRNA associated with exosomes, cell secreted lipoproteins can transport and deliver active miRNAs to distant cells [29, 101]. Previous studies have shown that the miRNA extracellular export process is regulated by the ceramide pathway [102]. Inhibition of the sphingomyelinase SMPD3 promoted miRNA release in association with HDL instead of exosomes. A similar regulation involving the ceramide pathway could occur particularly under physiological conditions where YRNAs are overly abundant in the exosome compartment.

To explain the unusual abundance of YRNAs in plasma-derived exosomes of our different Ugandan cohorts, we used quantitative mass spectrometry to characterize the exosome proteome. Studies have shown that the presence and abundance of exosomal proteins is reflective of the cellular source of the exosomes. In addition, the exosomal proteome can also reflect cellular changes due to infectious diseases or cancer [103]. In our study, the quantitative proteomic analysis of the exosomal proteome revealed an abundance of peptides from proteins usually expressed in extracellular vesicles released by erythrocytes and keratinocytes. The oral cavity has been identified as the major site for the shedding of infectious KSHV viral particles. Tonsillar human oral keratinocytes are believed to be the main source of virus in saliva since differentiated keratinocytes are permissive to KSHV infection and KSHV does not appear to replicate in salivary glands [6]. Interestingly, miR-26a-5p, the most abundant miRNA in plasma exosomes from our cohorts, is the only cellular miRNA whose level of expression in plasma correlated with KSHV genome copy number in oral swabs. These observations suggest that KSHV infected oral keratinocytes could contribute to the release of exosomes containing miR-26a-5p in the blood circulation.

In recent studies, patients infected with either plasmodium falciparum or plasmodium vivax showed elevated plasma levels of extracellular vesicles derived from platelets and erythrocytes [104, 105]. Of note, the extracellular vesicles released from Plasmodium -infected erythrocytes are referred to as exosome-like vesicles because they share similar size and features with exosomes released by other cell types. Using mass spectrometry, we detected an infection with malaria in our study participants by identifying several peptides associated with different proteins from Plasmodium falciparum, the parasite responsible for malaria in Uganda. Previous studies on exosomes-like vesicles released in plasma following exposure to malaria have mostly been performed in the context of patients with severe disease. Our study showed that extracellular vesicles circulating in plasma from individuals with asymptomatic malaria infection have particular genetic and protein contents that can potentially be used as diagnostic biomarkers. Our data suggests that the abundant accumulation of YRNAs in exosomes in plasma of individuals infected with Plasmodium alters the level of both cellular and viral exosomal miRNAs, limiting the diagnostic possibilities for miRNAs in such individuals.

An abundance of YRNA in exosomes was previously observed in chronic lymphocytic leukemia (CCL) [106]. An abundance of circulating YRNA fragments has also been observed in blood samples from patients with coronary artery disease [107]. Small RNA sequencing revealed that the YRNA4 (HY4) subtype was the most abundant small RNA species in plasma exosomes from CCL patients. Importantly, plasma exosomes from CCL patients promoted disease development and progression by enhancing tumor growth and stimulating angiogenesis in the early onset of the disease. It has been shown that either plasma exosomes purified from CCL patients or YRNA4 (HY4) RNA alone could trigger monocytes activation via the Toll-like receptor (TLR) 7/8 signaling pathway [63, 108]. Our data show that the RYNA4 (HY4) species is also the most abundant small RNA type in exosomal vesicles circulating in plasma from individuals with apparently uncomplicated or asymptomatic malaria infection. As such, our observations support a model in which processed fragments of YRNA4 (HY4) in exosome-like vesicles released by erythrocytes infected with malaria could mediate activation of distant monocytes by stimulating the TLR7/8 signaling pathway. Interestingly, activation of the TLR7/8 signaling pathway can also induce KSHV reactivation from latency. Hence, malaria infection could results in an increase in KSHV reactivation rates in co-infected individuals and so, account for the association observed between KSHV sero-positivity and malaria exposure in Ugandan people [109].

In geographic regions where malaria is endemic, many infected adults are asymptomatic with very low or undetectable parasitemia. These individuals constitute a potential reservoir of malaria parasites and a possible source of transmission to mosquitoes [110]. There is currently no high throughput screening method to diagnose malaria infection in these asymptomatic individuals. Extensive characterization of the plasma proteome by mass spectrometry has led to the discovery of several cellular proteins with the potential to be diagnostic biomarker for malaria infection and disease severity [72]. Nonetheless, the proteome associated with exosomal vesicles released in plasma from individuals exposed to malaria is still poorly defined. Using a quantitative proteomic approach, we identified peptides associated with 11 different proteins from *Plasmodium falciparum* in in plasma exosomes of both KSHV+/HIV- and KSHV+/HIV+ individuals. With the exception of PF3D7_0831700, an HSP70-like plasmodium protein, none of these proteins have previously been detected in blood samples from patients diagnosed with malaria. As such, these malarial proteins could be used as new plasma biomarkers to diagnose individuals with asymptomatic or uncomplicated malaria infection. Unfortunately, no data on parasitemia or hemoglobin blood levels were collected for any individuals enrolled in our study. Hence, we could not establish an association between disease stage and the relative level of malarial peptides in plasma exosomes. Previous proteomic studies identified several plasma proteins involved in the complement cascade, hemostasis and inflammatory response as promising biomarkers for malaria infection [69, 111, 112]. Our study showed that the majority of these proteins are also present in plasma exosomes of individuals infected with *Plasmodium falciparum*. Interestingly, the relative abundance of the peptides associated with the malarial proteins PH3D7_1239800, PH3D7_1460800, and PH3D7_0802700 strongly correlated with the relative abundance of peptides derived from several cellular proteins involved in the complement cascade, coagulation and inflammation. Although the function of the malarial proteins identified in plasma exosomes is unknown, their association with biomarkers of malarial disease suggests an important role in the parasite infection. Since the Ugandan individuals both in the KSHV+/HIV- and KSHV+/HIV+ groups had no overt signs of ongoing malaria disease during the study enrollment, the presence of these peptides could be a critical biomarker for asymtomatic *Plasmodium falciparum* infection with submicroscopic parasitemia [110]. Currently, infections with a low level of parasitemia can only be detected by molecular methods including PCR and more recently, Loop-mediated Isothermal Amplification (LAMP). These molecular diagnostic techniques have high screening costs and require basic laboratory infrastructures. In addition, malaria infections of very low density are still undetectable by PCR [113]. The malarial peptides identified in exosomes could allow for the development of a rapid diagnostic test to identify asymptomatic submicroscopic malaria infections.

Studies have shown that the complement cascade is activated during malaria infection [111]. Our data showed that in plasma exosomes of individuals infected with *Plasmodium falciparum*, the relative abundance of peptides associated with cellular proteins involved in both the classical (C4BP, C1QA) and alternative pathway (Properdin) of complement activation strongly correlated with the relative abundance of a peptide associated with the malarial protein PH3D7_1239800. Our data showed that proteins associated with the complement activation pathways can be found in exosomal vesicles circulating in plasma indicating that complement activation persists in infected individuals as observed previously. Previous studies have shown that KSHV-infected endothelial cells are more resistant to complement mediated cell lysis and that complement activation promotes infected cell survival by inducing STAT3 phosphorylation [114]. Interestingly, complement activation has also been observed in KS tumors [114]. Hence, persistent complement activation in KSHV-infected individuals exposed to malaria could create a more favorable environment for KSHV associated carcinogenesis by supporting inflammation and angiogenesis. Of note, complement activation is also observed following HIV infection [115] and can mediate HIV binding to erythrocytes [116] which is associated with increased viral infectivity [117].

Overall, our study revealed that the extracellular miRNA expression profile in plasma from individuals with a poly-microbial infection is the result of a dynamic process influenced by each infecting pathogen. Hence, the potential regulatory activity of the repertoire of extracellular miRNAs circulating in plasma is determined by the combined effects of the different pathogens. Our study suggests that polymicrobial infections could influence the process of exporting and releasing miRNAs into delivery vesicles such as exosomes or lipoproteins, making them difficult to use for diagnosis of individual pathogen infections. Our study also showed that therapeutic targeted at one pathogen, such as ART, can also alter the extracellular repertoire of host miRNAs ultimately playing a role in the outcome of gene regulation. Thus, therapeutic strategies for disease eradication should rely on a syndemic approach involving monitoring multiple endemic pathogens and developing treatments that consider potential effects on the biology and pathology of the different pathogens.

## Material and methods

### Study participants

The study was conducted at the Uganda Cancer Institute (UCI), Mulago Hospital, Makerere University, in Kampala, Uganda. KSHV and HIV viral status was determined for each patient. Blood samples were drawn on days 1, 7, and 15 and plasma was isolated by centrifugation. Plasma samples with visible sign of hemolysis were discarded. People were identified as KSHV-positive if KSHV DNA was detected by a specific qPCR assay at least twice in their oral swabs during the shedding period with a genome copy number >150copies/ml. Previous studies determined that KSHV-infected individuals have a high probability to test positive for KSHV DNA at least once during this length of time [7]. HIV negative or positive serostatus was evaluated using a point-of-care test that detects both HIV-1/2 antibodies and free HIV-1 p24 antigen (Determine HIV-1/2, Alere Waltham, MA). Positive results were confirmed in a second rapid chromatographic assay detecting antibodies to Human HIV-1 and HIV-2 (Clearview Stat-Pak, Alere). Only HIV positive participants who had a CD4+ T-cell count above 200/mm3 and were not taking antiretroviral medication (ART) at the time of enrollment were eligible for the study. None of the study participants was diagnosed with Kaposi’s sarcoma. The enrollment protocol did not included screening for malaria infection by specific rapid diagnostic test or blood smear. Patients with an asymptomatic or uncomplicated malaria infection would have met all criteria for eligibility.

KSHV viral status was determined based on KSHV genome detection by specific qPCR in either oral swabs or plasma samples at least once during a period of 15 consecutive days. Oral swab collection and processing were performed as previously described [10]. DNA was extracted from mucosal swabs and plasma and KSHV DNA was measured by qPCR with primers to the orf73 gene, with positive and negative controls as previously described.

### Ethics statement

All participants gave informed consent for study participation. Ethical approval for all study procedures was obtained from the Makerere University Research and Ethics Committee, the Uganda National Council for Science and Technology, Seattle Children’s Hospital, Fred Hutchinson Cancer Research Center and the University of Washington Human Subjects Division. All subjects were adults and provided informed consent. For literate study participants, the informed consent given was written. For illiterate study participants, the informed consent given was oral as approved by the IRB. In this case a literate witness was required to be present to document the oral consent given by the participant.

### NanoString nCounter system miRNA assay

Total RNA was isolated from 1 ml of plasma using the RNAzol kit (Zymmoresearch Irvine CA) according to manufacturer protocol. The samples were prepared for nCounter miRNA expression profiling according to the manufacturer’s recommendations (NanoString Technologies, Seattle WA). The repertoire of cellular miRNAs present in plasma was analyzed using the sample obtained on Day 0 of the screening period. For each sample, a scan of 600 fields of view (FOV) was imaged. Normalization of raw data was performed using the nSolver software 3.0 (NanoString Technologies Seattle WA). Background substraction was performed using the mean value of the negative controls. NanoString counts were normalized for all target miRNAs by using the top 100 robustly expressed miRNAs. Only miRNAs with field of view (FOV) values >100 for all individuals from at least one cohort were considered for differential expression analysis. Data collected from groups of individuals from all three cohorts were then analyzed using the Kruskal-Wallis statistical test to determine miRNAs with a significant differential expression (P ≤ 0.05). MiRNAs were defined as “ongogenic” or “tumor suppressor” based on a classification previously reported in a publication on miRNAs expression in KSHV associated AIDS malignancies [11]. In some cases, the function associated with miRNA expression was deduced from compiled published observations available on the mircancer website (mircancer.ecu.edu)

### Total small RNA isolation from plasma

1 mL of plasma was filtered through 0.45 μm filters (Costar SpinX Fisher Scientific, Hampton NH) and RNA was then isolated from the samples in 3 aliquots of 300 μl cleared serum utilizing TRI Reagent BD (Molecular Research Center, Cincinnati, OH). After phase separation, the upper phase was combined with 100% ethanol, purified using RNA silica spin columns (EPOCH Life Sciences, Missouri City, Texas), and eluted in RNase-free water. The eluate from all 3 isolation tubes were pooled, re-extracted with TRI Reagent (Molecular Research Center, Cincinnati, OH), and re-precipitated.

### Exosomal small RNA isolation

A total of 1 mL of plasma was cleared of cellular debris by centrifugation at 3,000 g for 15 minutes at 4°C. The RNA was then isolated using the Qiagen exoRNeasy Serum/Plasma Midi Kit (Qiagen Germantown, MD). The RNA was then digested with RNase-free DNase I (Epicentre, Madison, WI) and re-purified on RNeasy MinElute columns (Qiagen Germantown, MD).

### Small RNA isolation quality control

To determine the effectiveness of RNA isolation, each sample was screened for cel-miR-257, hsa-miR-150, and hsa-miR-451 by specific RT-qPCR. MiR-257 was added as a synthetic microRNA at a ratio of 7.5 fmoles/ml of plasma early in the isolation process. The calculated value of Relative Quantity (RQ) per fmole of miR-257 following efficient RNA isolation should be 1 or greater in each sample. In addition, expected CT values for the two endogenous microRNAs (miR-150 and miR-451) should be in the detectable range (CT < 35).

### Library preparation

Template DNA molecules suitable for cluster generation were prepared from the isolated RNA samples using the NEBNext Small RNA-Seq Library Preparation Kit (New England BioLabs, Ipswich, MA) according to the manufacturer’s instructions, except that following the RNA linker ligation, and after cDNA synthesis, the single stranded cDNA was fractionated using a 12% acrylamide-urea gel. cDNA fragments of 57–88 nucleotides (nt) corresponding to inserts of 11–41 nt were excised and purified, in order to ensure that molecules with 17–35 nt inserts were recovered. Following the NEBNext protocol, the purified libraries were electrophoresed through a freshly cast 6% native acrylamide gel and library fragments of 128–146 nt corresponding to inserts of 11–29 nt (fragment of 128–158 nt corresponding to inserts of 11–41 nt in case of exosome samples) were excised from the gel and recovered by overnight agitation at 37°C at 200 RPM in elution buffer, passage of the eluate through a 0.45 μm filter, and ethanol precipitation. The quality and size distribution of the amplified libraries were determined using Agilent 2100 Bioanalyzer High Sensitivity DNA microfluidic chips (Agilent Technologies, Santa Clara, CA). Libraries were quantified using the KAPA Library Quantification Kit (Kapa Biosystems-Roche, St. Louis MO).

### High throughput sequencing

The libraries were pooled at equimolar concentrations and diluted prior to loading onto an Illumina HiSeq Flow Cell v4 on the Illumina cBot cluster station (Illumina Inc. San Diego, CA). The libraries were extended and bridge amplified to create single sequence clusters using the HiSeq SR Cluster Kit v4 cBot. The flow cell carrying amplified clusters was loaded on the HiSeq 2500 sequencing system and sequenced with 50-nt single-end reads using the HiSeq SBS Kit v4. 10% ΦX174 phage DNA was spiked in all sequencing lanes for sequencer calibration. Real time image analysis and base calling were performed on the instrument using the HiSeq Sequencing Control Software v2.2.58. Illumina bcl2fastq v1.8.3 software was used for demultiplexing and production of FASTQ sequence files.

### Data processing

The FASTQ sequence files were merged for each sample and then filtered to remove reads that were flagged by Illumina software as low quality. The FASTX (http://hannonlab.cshl.edu/fastx_toolkit/) application was used to trim the 3’-end of sequence reads in order to remove the 3’ adaptor sequence (AGATCGGAAGAGCACACGTCTGAACTCCAGTCAC); sequences without any 3’ adapter sequence as well as sequences of less than 17 nucleotides after trimming were removed. A Perl script was used to remove sequences with any amount of 5’-adapter sequence (AATGATACGGCGACCACCGAGATCTAC ACG TTCAGAGTTCTACAGTCCGACGATC). FASTX was also used to collapse identical reads into single entries retaining the read count for each unique sequence. To filter out any sequencing errors, only sequence reads that occurred at least 5 times were retained for further analysis. Non-redundant sequences were then aligned to genomic and mRNA sequences using bowtie 2 with hg19 as reference genome; sequences with perfect-match and 1bp mismatch alignments were retained for further analysis.

The mapped sequences were aligned to common and abundant non-coding RNAs (tRNAs, rRNAs, YRNAs and snoRNAs (http://www-snorna.biotoul.fr/index.php), again using bowtie 2 software (refLangmeat). The genome mapped sequences were further aligned to precursor and mature microRNAs in miRBase 21.0 (ref mirbase Kosomara) and piRNAs from a piRNA database (ref rosenkranz) using an alignment software. Bowtie 2 was also used to align mapped sequences to the Ensembl 80 cDNA database for transcripts.

### Principal component analysis (PCA)

PCA analysis was performed separately using the module built in to the National Institute of Ageing (NIA) Array Analysis software. Parameters used were as followed: covariance as the matrix type, 3 principal components, 1.5 as the fold change threshold for clusters, 0.7 as the correlation threshold for clusters, and all microRNAs to analyze and display.

### Hierarchical cluster analysis

Comparison between miRNA expression profiles from KSHV-infected individuals with or without HIV was performed using hierarchical cluster analysis (CIMminer). The Euclidian distance matrix was computed based on the respective normalized log value calculated for each miRNA predicted to be differentially expressed in the different plasma specimens. These values were used as input for hierarchical clustering using the complete linkage-clustering algorithm. Data sets representing the viral gene expression profiles were visualized by heat maps using color-coded Clustered ImageMaps software (CIMminer) [118].

### Statistical analysis

Statistics was performed using Graphpad Prism version 6.01 for Windows (GraphPad Software). Comparison between cellular miRNA levels of expression in patient prior or following ART therapy was performed using a ratio paired t test on logarithm transformed FOV values. Differential expression analysis on the RNAseq data was performed using DEseq2 software [119]. To compare the overall read numbers of viral or cellular miRNAs with either KSHV rate of shedding, rate of viremia and KSHV oral or plasma viral loads, two-tailed Spearman nonparametric correlation coefficients (r2) and corresponding P-values were calculated using 95% confidence intervals. A Spearman correlation test was also used to compare relative abundances between peptides from malarial proteins and peptides associated with cellular plasma proteins.

### Proteomics analysis

#### Sample preparation and protein fractionation

Exosomes were enriched from plasma samples using the exoquick-LP kit to reduce contamination by lipoprotein particles prior to precipitation of exosomes. The quality of the purified exomes was confirmed by electron microscopy. Exosome pellets were re-suspended in RIPA buffer and the total protein amount was determined by bicinchoninic acid colorimetric assay (BCA) [120] (Thermo Fisher, Waltham, MA). 30 μg of protein was precipitated from each sample using acetone at -20°C overnight. After centrifugation for 20 minutes at 15,000×g, acetone was decanted, and the air dried protein pellets were dissolved in 30 μL of 8 M urea. Each sample was reduced by adding 15 μL of 10 mM dithiothreitol (DTT) and 100 mM ammonium bicarbonate in water and then incubated for 45 min at 56°C. The cysteine residues were alkylated by adding 9 μL of 50 mM iodoacetamide and 100mM ammonium bicarbonate in water to each vial and then incubating for 30 minutes in darkness. An additional 9 μL of 10 mM DTT and 100mM ammonium bicarbonate in water was added to neutralize the iodoacetamide and all solutions were diluted with 50 mM ammonium bicarbonate to 1M urea concentration. All samples were trypsinized by adding 1 μg of trypsin (Promega, Madison, WI) in 10 μL of 50 mM ammonium bicarbonate to each and incubating them overnight at 37°C. Each sample was diluted further with 0.1% trifluoroacetic acid (TFA) to 800 μL and cleaned using Oasis HLB 10mg cartridges (Waters, Milford, MA) separately. The cartridges were conditioned with 1 mL acetonitrile, 2 × 1 mL 65% acetonitrile 0.1% TFA in water, and 2 × 1 mL 0.1% TFA. After sample loading in 1 mL of 0.1% TFA and washing 2 × 1 mL 0.1% TFA, peptides were eluted using 1 mL of 65% acetonitrile and 0.1% TFA in water and dried in a speed vac. Each sample was then labeled with a unique tag allowing for differential quantification. The dried samples were dissolved in 100 μL of 200mM HEPES. One of 10 TMT™ mass tag labeling reagents, 0.8mg (Thermo Scientific, Waltham, MA) was then added in 40 μL of acetonitrile to each vial, and the 10 vials were incubated at room temperature for 1 hour. The labeling reaction was quenched by adding 8 μL of 5% Hydroxlamine in water to each vial. All vials were dried in a speed vac separately. The labeled peptides from 10 samples were dissolved in 50 μL 0.1% TFA in each vial and combined. The pH was adjusted to less than 3 with 2% TFA right before desalting using Oasis HLB 10mg cartridge (Waters, Milford, MA) and stored at -20°C until fractionation by strong cation-exchange (SCX) chromatography.

The pooled sample was dissolved in buffer A (7 mM potassium phosphate, 30% acetonitrile, pH 2.65) immediately before fractionation with a SCX column (PolySulfoethy A, 5 μm, 2.1 × 100 mm, PolyLC Inc., Columbia, MD) The fractions were collected every minute at a flow rate of 200 μL/min from 1% solvent B (7 mM potassium phosphate, 500 mM KCl, 30% acetonitrile, pH 2.65) to 60% over 40 minutes (1% B for 7 minutes, 6–15% B for 18 minutes, 15–34% B for 10 minutes, and 34–60% B for 5 minutes) as well as during column washing with 98% solvent B for 10 minutes. The peptide elution was monitored using a UV detector at λ = 220 nm. These fractions were consolidated into 10 fractions using the UV trace to evenly distribute the peptide quantities. After drying in a speed vac, peptides were desalted using Oasis HLB 10mg cartridges (Waters, Milford, MA), and dried in a speed vac.

#### LC-MS/MS analysis

LC-MS/MS analysis was performed with an Easy-nLC 1000 (Thermo Scientific) coupled to an Orbitrap Fusion mass spectrometer (Thermo Scientific Waltham, MA). The LC system configured in a vented format consisted of a fused-silica nanospray needle (PicoTip™ emitter, 75 μm ID, New Objective, Woburn, MA) packed in-house with Magic C18 AQ 100Å reverse-phase media (Michrom Bioresources Inc., Auburn, California) (26 cm), and a trap (IntegraFrit™ Capillary, 100 μm ID, New Objective Woburn, MA) containing Magic C18 AQ 200Å (2 cm). Each fraction was dissolved in 40 μL of 2% acetonitrile and 0.1% formic acid in water, and 1–5 μL was loaded onto the column and separated using a two-mobile-phase system consisting of 0.1% formic acid in water (A) and 0.1% formic acid in acetonitrile (B). The chromatographic separation was achieved over a 107-min gradient from 3% to 50% B (3–10% B for 5min, 10–33% B for 90 min, 33–50% B for 7 min, and 50% B for 5 min) at a flow rate of 400 nL/min. The mass spectrometer was operated in a data-dependent MS/MS mode over the m/z range of 400–1500. The mass resolution was set to 120,000. The cycle time was set to 3 seconds, and the most abundant ions from the precursor scan were isolated for MS/MS analysis using a quadrupole with 1.6 (*m/z*) mass window, dissociated with 40% normalized HCD collision energy and analyzed with an orbitrap with the resolution set to 60,000. Selected ions were dynamically excluded for 30 seconds.

#### Peptide and protein identification from mass spectra of digested fragments

Proteome Discoverer™ version 2.1 was used for data normalization and analysis. The MS/MS data were searched against the Uniprot human proteome database, common contaminants (http://www.thegpm.org/crap/), Uniprot HIV 1 and NCBI human herpes virus -4,-6,-7,-8, cytomegalovirus (CMV) and plasmodium falciparum (strain 3D7) using SEQUEST (Eng et al). The following parameters were used: trypsin as the digestion protease; precursor and fragment error tolerance at 10 parts per million (ppm) and 0.6 Da; TMT modification of N-termini as a fixed modification; and TMT modification of lysine residues, carbamidomethyl on cysteine residues, and oxidation of methionine residues as variable modifications. Identified peptides were filtered by a 1% peptide-level false discovery rate (FDR) using q-value of 0.01 from Percolator [121].

## Supporting information

S1 Table Normalized nanostring data obtained using the nCounter miRNA expression assay. Normalization of raw data was performed using the nSolver software 3.0 (NanoString Technologies Seattle WA). Background substraction was performed using the mean value of the negative controls. NanoString counts were normalized for all target miRNAs by using the top 100 robustly expressed miRNAs. MiRNAs with a significant change in level of expression when comparing the groups of individuals pairwise are shown in panel B. P values indicate were calculated using a t-test. A classification for each miRNA expected activity and the percentage of oncogenic and tumor suppressor miRNAs significantly altered for each cohort is shown. MiRNAs for which no previously published observations are available to allow for a classification are listed as “NA”.
(XLSX)

S2 Table Hosts plasma proteins unique to plasma exosomes from patients infected with malaria. List of all the classes of proteins identified by mass spectrometry that do not overlap with the extracellular vesicle proteome from the different databases tested.
(XLSX)
